# Engineering *Streptomyces coelicolor* for heterologous expression of the thiopeptide GE2270A—A cautionary tale

**DOI:** 10.1093/jimb/kuaf019

**Published:** 2025-07-18

**Authors:** Francesco Del Carratore, Erik K R Hanko, Kamila Schmidt, Oksana Bilyk, Suhui Ye Huang, Marianna Iorio, Mercedes Pérez-Bonilla, Rosario Pérez-Redondo, Michelle Rudden, Emmanuele Severi, Arianna Tocchetti, Margherita Sosio, Emily J Johnson, Timothy Kirkwood, Dominic R Whittall, Alkisti Manousaki, Olga Genilloud, Antonio Rodríguez-García, Gavin H Thomas, Stefano Donadio, Rainer Breitling, Eriko Takano

**Affiliations:** Department of Biochemistry, Cell, and Systems Biology, Institute of Integrative, Systems and Molecular Biology, University of Liverpool, Liverpool L69 3BX, UK; Manchester Institute of Biotechnology, School of Chemistry, Faculty of Science and Engineering, University of Manchester, Manchester M1 7DN, UK; Manchester Institute of Biotechnology, School of Chemistry, Faculty of Science and Engineering, University of Manchester, Manchester M1 7DN, UK; Manchester Institute of Biotechnology, School of Chemistry, Faculty of Science and Engineering, University of Manchester, Manchester M1 7DN, UK; Division of Immunology, Immunity to Infection and Respiratory Medicine, School of Biological Sciences, Faculty of Biology, Medicine and Health, University of Manchester, Oxford Road, Manchester M13 9PL, UK; Manchester Institute of Biotechnology, School of Chemistry, Faculty of Science and Engineering, University of Manchester, Manchester M1 7DN, UK; Manchester Institute of Biotechnology, School of Chemistry, Faculty of Science and Engineering, University of Manchester, Manchester M1 7DN, UK; Área de Microbiología, Departamento de Biología Funcional, Universidad de Oviedo, Oviedo, Principality of Asturias, Spain; NAICONS Srl, Milan, Italy; Fundación MEDINA, Parque Tecnológico Ciencias de la Salud, Avda. Conocimiento 34, 18016 Granada, Spain; Instituto de Biotecnología de León, INBIOTEC, León, Spain; Fundación Centro de Servicios y Promoción Forestal y de su Industria de Castilla y León, Cesefor. Sede INBIOTEC, Avda. Real 1, 24006 León, España; Department of Biology and York Biomedical Research Institute (YBRI), University of York, Wentworth Way, York YO10 5DD, UK; Department of Biology and York Biomedical Research Institute (YBRI), University of York, Wentworth Way, York YO10 5DD, UK; Department of Applied Sciences, Ellison Building, Northumbria University, Ellison Place, Newcastle-Upon-Tyne NE1 8ST, UK; NAICONS Srl, Milan, Italy; NAICONS Srl, Milan, Italy; Computational Biology Facility, Liverpool Shared Research Facilities, Faculty of Health and Life Sciences, University of Liverpool, Liverpool L69 7ZB, UK; Manchester Institute of Biotechnology, School of Chemistry, Faculty of Science and Engineering, University of Manchester, Manchester M1 7DN, UK; Manchester Institute of Biotechnology, School of Chemistry, Faculty of Science and Engineering, University of Manchester, Manchester M1 7DN, UK; Manchester Institute of Biotechnology, School of Chemistry, Faculty of Science and Engineering, University of Manchester, Manchester M1 7DN, UK; Fundación MEDINA, Parque Tecnológico Ciencias de la Salud, Avda. Conocimiento 34, 18016 Granada, Spain; Área de Microbiología, Departamento de Biología Molecular, Facultad de Ciencias Biológicas y Ambientales, Universidad de León, León, Spain; Instituto de Biotecnología de León, INBIOTEC, León, Spain; Fundación Centro de Servicios y Promoción Forestal y de su Industria de Castilla y León, Cesefor. Sede INBIOTEC, Avda. Real 1, 24006 León, España; NAICONS Srl, Milan, Italy; Manchester Institute of Biotechnology, School of Chemistry, Faculty of Science and Engineering, University of Manchester, Manchester M1 7DN, UK; Manchester Institute of Biotechnology, School of Chemistry, Faculty of Science and Engineering, University of Manchester, Manchester M1 7DN, UK

**Keywords:** Metabolic engineering, Synthetic biology, *Streptomyces coelicolor*, Thiopeptide, GE2270A

## Abstract

The thiopeptide GE2270A is a clinically relevant, ribosomally synthesised, and post-translationally modified peptide naturally produced by *Planobispora rosea*. Due to the genetically intractable nature of *P. rosea*, heterologous expression is considered a possible route to yield improvement. In this study, we focused on improving GE2270A production through heterologous expression of the biosynthetic gene cluster (BGC) in the model organism *Streptomyces coelicolor* M1146. A statistically significant yield improvement was obtained in the *S. coelicolor* system through the data-driven rational engineering of the BGC, including the introduction of additional copies of key biosynthetic and regulatory genes. However, despite our best efforts, the highest production level observed in the strains generated in this study is 12× lower than published titres achieved in the natural producer and 50× lower than published titres obtained using *Nonomuraea* ATCC 39727 as expression host. These results suggest that, while using the most genetically amenable strain as host can be the right choice when exploring different BGC designs, the choice of the most suitable host has a major effect on the achievable yield and should be carefully considered. The analysis of the multiomics data obtained in this study suggests an important role of PbtX in GE2270A biosynthesis and provides insights into the differences in production metabolic profiles between the different strains.

**One Sentence Summary:** Data-driven rational engineering of *Streptomyces coelicolor* for heterologous production of the thiopeptide antibiotic GE2270A resulted in increased production but encountered unexpected challenges compared to production in the natural producer or the alternative host *Nonomuraea* ATCC 39727.

## Graphical Abstract

**Graphical Abstract:**
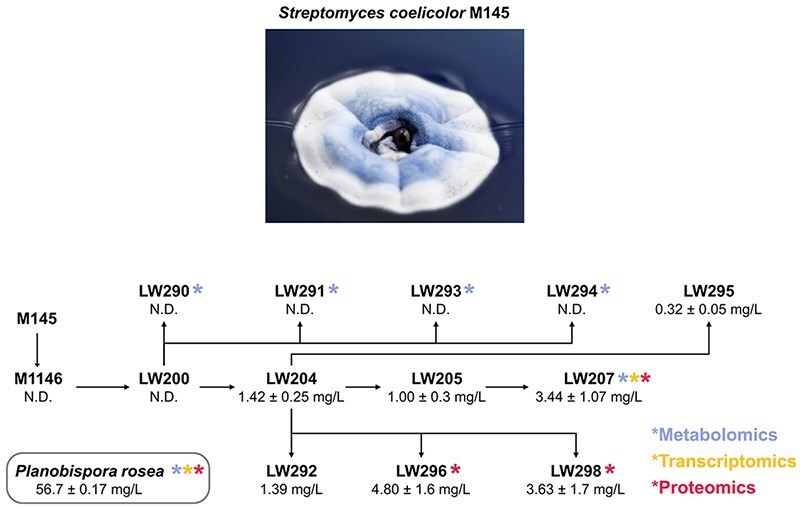

## Introduction

**Fig. 1.**
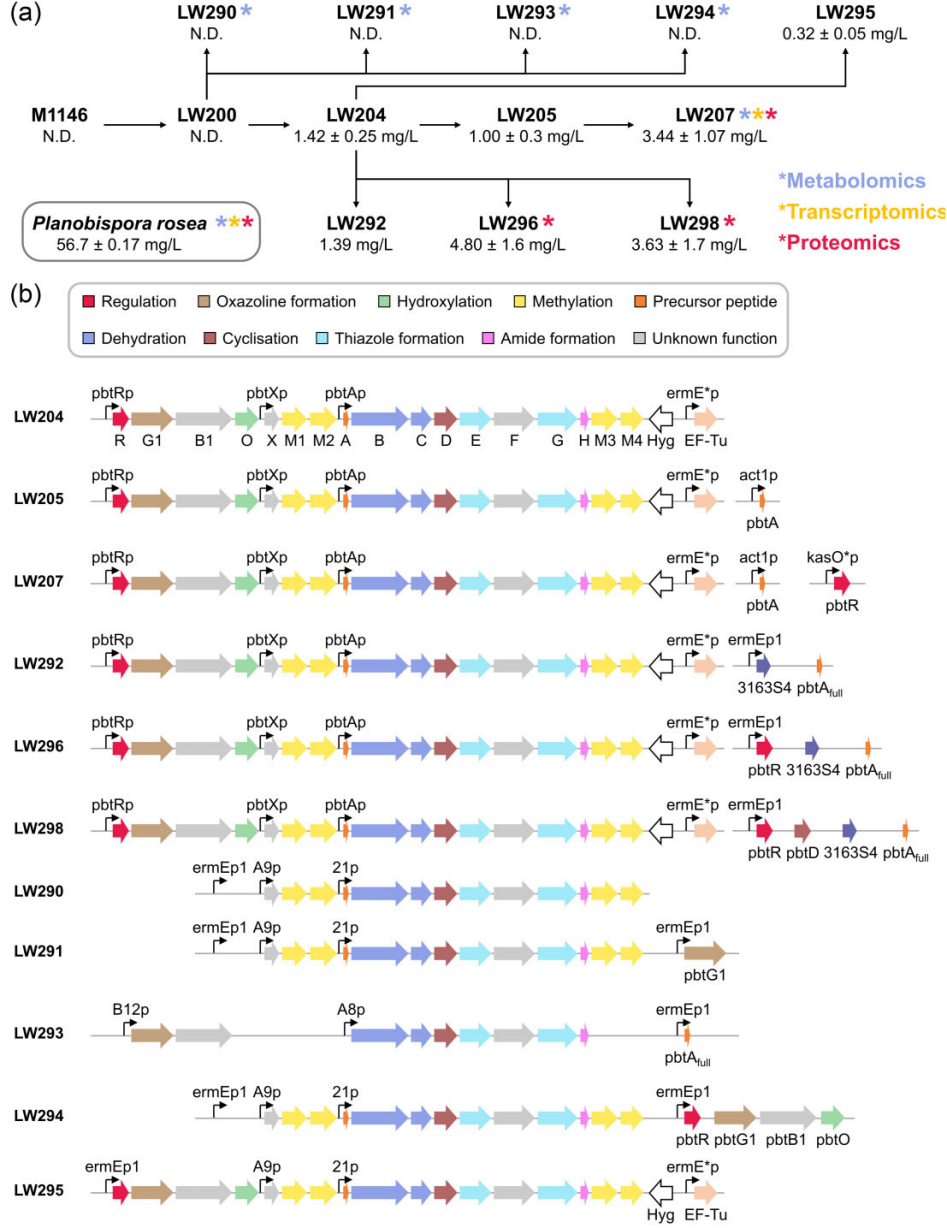
Summary of strains generated in this study. (A) Pedigree chart for the *Streptomyces coelicolor* strains used and generated in this study. Strains for which metabolomics, transcriptomics, and proteomics data were collected are denoted by asterisks. GE2270A titres are indicated for each strain (mean ± standard deviation). N.D.—not detected. Titres represent means of biological replicates (*N* = 2–9). (B) The strains outlined in A differ from each other only in the GE2270A BGC. Differences are illustrated here graphically. Promoters are indicated by arrows and protein functions are colour coded. Gene names are annotated in LW204, containing the unmodified version of the GE2270A cluster, including the hygromycin selection marker (Hyg) and the elongation factor TU (EF-Tu).

GE2270A is a thiopeptide antibiotic that has potent activity against Gram-positive pathogens through binding and inhibiting the function of elongation factor Tu (EF-Tu) (Selva et al., 1991). This ribosomally synthesised and post-translationally modified peptide (RiPP) is regarded as a potentially clinically relevant natural product as it is used as a precursor for the semisynthesis of two compounds, LFF-571 and CB-06-01, also known as NAI003. Although their development has been halted for commercial reasons, LFF-571 has been developed as an antibiotic for *Clostridioides difficile* infections (Butler et al., 2017; LaMarche et al., 2011), while CB-06-01 has completed a Phase I clinical study as a topical treatment for acne (Butler, 2008; Donadio et al., 2010; Fabbretti et al., 2015). As is the case for all RiPPs, the biosynthesis of GE2270A begins with the synthesis of a precursor peptide (consisting of leader and core peptide) encoded by the *pbtA* gene. Subsequently, the precursor peptide undergoes several modifications of the core peptide region, and the leader peptide is then proteolytically cleaved. Finally, the resulting modified core peptide, that is the mature RiPP, is exported from the producing cell (Arnison et al., 2013); however, the export mechanism for this specific compound has not been characterised yet. GE2270A is naturally produced by *Planobispora rosea* and *Nonomuraea* sp. WU8817 (Morris et al., 2009), two bacteria belonging to the Streptosporangiaceae family of Actinomycetota. Both have been used to produce GE2270A on an industrial scale, and a recent multiomics study on the fermentation of *P. rosea* provided a comprehensive understanding of its metabolism (Del Carratore et al., 2021). A rational optimization of the natural producers is prevented by their genetic intractability. This limitation could potentially be circumvented through the heterologous expression of the biosynthetic gene cluster (BGC) of GE2270A in a more amenable organism. In fact, the heterologous expression of this BGC has been successful in both *Streptomyces coelicolor* M1146 (Flinspach et al., 2014) and in *Nonomuraea* ATCC 39727 (Tocchetti et al., 2013). Unlike *Nonomuraea* sp. WU8817, *Nonomuraea* ATCC 39727 (also known as *Nonomuraea gerenzanensis*), is a different species of the genus *Nonomuraea* that is not a native producer of GE2270A, but is genetically tractable (Marcone et al., 2010). Recent advances in molecular biology and genetic engineering have allowed the use of *Streptomyces* species, and specifically the model organism *S. coelicolor*, as very successful cell factories for the heterologous expression of natural products (Del Carratore et al., 2022). Heterologous production of GE2270A was previously observed in *S. coelicolor* M1146 after screening several fermentation media and only at very low levels. In addition, the strain also exhibits sensitivity to this molecule (Flinspach et al., 2014). In this study, we aimed to obtain a *S. coelicolor* strain resistant to GE2270A and to use this strain to improve the GE2270A yield through a data-driven rational refactoring of the BGC (Fig. 1). This approach led to a statistically significant and reproducible yield increase, but production levels remained very modest in absolute terms compared to those obtained in the natural producer *P. rosea*. Much higher yields were obtained when expressing the same constructs in a different host (*Nonomuraea* ATCC 39727) (Tocchetti et al., 2013). While choosing the most genetically amenable strain as an expression host can be the right choice when exploring different BGC designs, exploring different hosts should be considered when aiming for high yields.

## Materials and Methods

### Base Strains, Media, and Growth Conditions

*Streptomyces coelicolor* M1146 (Gomez‐Escribano & Bibb, 2011) and *Nonomuraea* ATCC 39727 (*gerenzanensis*) served as heterologous hosts for GE2270 biosynthesis. *Escherichia coli* 5ɑ (New England Biolabs, NEB) was employed for cloning and plasmid propagation. The strains *E. coli* ET12567/pUZ8002 and *E. coli* ET12567/pUB307 were used for the conjugative transfer of plasmids into *S. coelicolor* and *Nonomuraea* ATCC 39727, respectively (Bennett et al., 1977; Flett et al., 2006; MacNeil et al., 1992).

*Escherichia coli* strains were routinely grown in Lysogeny Broth (LB) medium at 37 °C, while *S. coelicolor* and *Nonomuraea* ATCC 39727 were cultivated at 30 and 28 °C, respectively. MSA (Kieser, 2000) was used for *S. coelicolor* transformation and sporulation, and LB was employed for spore quantification. Thiopeptide antibiotic toxicity assays were conducted in tryptone soy broth (TSB). For GE2270 biosynthesis in derivatives of *S. coelicolor* M1146, seed cultures were cultivated in TSB, and main cultures were grown in freshly prepared CMan medium. CMan medium comprised 10 g/L anhydrous d-glucose (Fisher Scientific, 10 141 520), 35 g/L soluble starch (Alfa Aesar, A11961), 5 g/L hydrolysed casein (Sigma-Aldrich, 22 090), 8 g/L yeast extract (Thermo Scientific, 212 750), 2 g/L CaCO_3_ (Sigma-Aldrich, 21 069), adjusted to a pH of 7.2 using KOH. For *Nonomuraea* ATCC 39727 transformation, RARE3, MV0.1X agar, and BTT agar were employed (Stinchi et al., 2003; Tocchetti et al., 2013). Antibiotics were added when needed at the following concentrations: 50 µg/mL kanamycin, 25 µg/mL nalidixic acid, and 15, 50, or 20 µg/mL apramycin for *E. coli, S. coelicolor*, or *Nonomuraea* ATCC 39727, respectively.

### Cloning and Transformation

Plasmid DNA was extracted using the QIAprep Spin Miniprep Kit (Qiagen, Hilden, Germany). DNA for cloning was amplified by PCR in 50 µL reactions using the Q5 High-Fidelity 2X Master Mix (NEB, Hitchin, UK). Gel-purified linearised DNA was purified using the QIAquick Gel Extraction Kit (Qiagen, Hilden, Germany). The NEBuilder HiFi DNA Assembly Master Mix, restriction enzymes, and T4 DNA ligase were purchased from NEB. PCR-, digestion-, and ligation reactions were set up according to the manufacturer's protocol. *Escherichia coli* was made competent through an established chemical method and transformed by heat shock (Sambrook et al., 1989). *Streptomyces coelicolor* and *Nonomuraea* ATCC 39727 were transformed by conjugation as reported previously (MacNeil et al., 1992; Stinchi et al., 2003). All strains used and generated in this study are summarised in Table S1.

### Plasmid Construction

All oligonucleotide primers were synthesised by Integrated DNA Technologies and are listed in Table S2. Plasmids were constructed by NEBuilder HiFi DNA assembly or conventional restriction enzyme-based cloning. Detailed assembly descriptions for all plasmids can be found in the Supplementary Methods. Correctly constructed plasmids were validated by Sanger sequencing. All plasmids used and generated in this study are listed in Table S3.

### Bioassays in Liquid Medium

For the liquid bioassays and minimum inhibitory concentration determination, 96-well plates were prepared by adding 50 μL of TSB containing 10⁵ colony forming units per well. Subsequently, 50 μL of TSB with the desired concentration of the pure compound was added to each well, following a series of 1/2 serial dilutions to achieve the required concentrations. The final volume in each well was adjusted to 100 μL. The plates were covered with a breathable sealing film to prevent contamination and evaporation. The sealed plates were then incubated for 48 hrs at 30 °C, 75% relative humidity, and 950 rpm in a shaking incubator (Infors HT; Multitron Pro; shaking throw: 3/12.5/25/50 mm).

### Production of GE2270

To produce GE2270 in derivatives of *S. coelicolor*, 50 mL of TSB was inoculated with 10^6^ spores in 250 mL shake flasks containing stainless steel coil springs (Coil Springs Direct Ltd.; Compression Spring 300 × 10 × 5 mm). Seed cultures were incubated for 48 hrs at 220 rpm and 30 °C. Main cultures were set up by inoculating 50 mL of CMan medium with 2 mL of the seed cultures in 250 mL shake flasks containing stainless steel coil springs. Cultures were returned to the shaker-incubator for 144 hrs.

### Quantification of GE2270

For the quantification of GE2270 in culture samples, 0.7 mL of the culture was transferred to a 2-mL Eppendorf tube and mixed with an equal volume of 100% acetonitrile (ACN). The mixture was agitated for 1 hr at room temperature and 60 rpm, centrifuged at 10,000× *g* for 2 min, and subsequently kept at −80 °C for 2 hrs to allow for phase separation. The organic phase was transferred to a separate tube and mixed with high-performance liquid chromatography (HPLC) grade water to achieve a final ACN:H_2_O ratio of 70:30.

GE2270 was quantified by HPLC using an Agilent 1260 Infinity II LC system equipped with a diode array detector measuring absorbance at 310 nm. For extract separation, a Poroshell 120 EC-C18 column (4 µm, 4.6 × 100 mm, Agilent) was used at 40 °C. The separation was achieved using a flow rate of 0.8 mL min^−1^ and a binary mobile phase consisting of A (H_2_O) and B (ACN, 0.1% formic acid). The gradient elution program was: 0–1 min, hold at 90% A; 1–9 min, 90–30% A; 9–11 min, 30–5% A; 11–12 min, 5–90% A; 12–14 min, hold at 90% A. The injection volume was 50 µL. GE2270 titres were quantified using calibration curves generated from running standards of known concentrations: 25, 12.5, 6.25, 3.125, 1.56, and 0.78 mg/L. Peak areas were integrated using Agilent OpenLab software.

### Sample Extraction for Metabolomics

Samples were collected in three biological replicates to determine either the exometabolome, the endometabolome or the whole-broth metabolite profile. For the whole-broth extraction, 1 mL of the culture was extracted by adding the same volume of ACN (ACN:H_2_O, 1:1) and mixed by vortexing. Aliquots of 200 μL were centrifuged for 10  min (4 °C, at 4,500 rpm) and dried in a Speedvac. The dried cell extracts were stored at −80 °C until Liquid Chromatography-Mass Spectrometry (LC-MS) analysis. For exometabolome analysis, 1 mL of culture medium was collected, centrifuged at 5,000× *g* for 10 min and then subjected to a flash freezing in liquid nitrogen for 1 min. After thawing (on ice), aliquots of 200 μL of the sample were dried in a Speedvac at room temperature. The dried samples were stored at −80 °C until LC-MS analysis. For endometabolome analysis, 10  mL of a cold (−48 °C) quenching solution (60% methanol) was added to 5 mL of bacterial culture, and the solution was centrifuged at 5,000× *g* for 10 min (4 °C). Next, the supernatant was discarded, and 1 mL of a cold (−48 °C) extraction solution (100% methanol) was added to the cell pellet, which was then transferred to an Eppendorf tube. Metabolites were extracted by three freeze-thaw cycles in liquid N_2_ (i.e. flash frozen in liquid N_2_ for 1 min, thawed on ice, and vortexed). Then the samples were centrifuged at maximum speed for 5 min (−9 °C), and 200 μL aliquots of supernatant dried in a Speedvac at room temperature. The dried cell extracts were stored at −80 °C until LC-MS analysis. On the day of the analysis, the samples were thawed and reconstituted in 200 μL of 20% methanol solution. The samples were vortexed, sonicated for 15 min, and analysed.

### LC-MS Metabolomics Data Acquisition

The cell extracts were analysed by Q Exactive Plus coupled to an Ultimate 3000 ultrahigh-performance liquid chromatography (ThermoFisher, Altrincham, Cheshire, UK) equipped with a Hypersil Gold C18 reversed-phase HPLC column (3 μm, 2.1 mm, 100 mm; catalogue no. 25003-102 130; ThermoFisher, UK). The mobile phase consisted of solvent A (H_2_O, 0.1% formic acid) and solvent B (methanol,  0.1% formic acid). The flow gradient was programmed to equilibrate at 95% solvent A for 2 min, followed by a linear gradient to 95% solvent B over 8 min, held at 95% solvent B for 2 min, then followed by a return to 95% solvent A in 0.25 min and held at 95% solvent A for a further 2 min. The column was maintained at 40 °C, and samples were chilled in the autosampler at 4 °C. The flow rate was set at 0.4 mL/min. The sample injection volume was 5 μL. Blank injections were analysed at the start and end of the analytical batch to assess the carryover. In addition, pooled quality control (QC) samples were analysed at every sixth injection to assess for analytical drift over time. The sample sequence was randomised. Data were acquired in full MS mode in the scan range of 90–1 350 m/z, with a resolution of 70,000, an AGC target of 3e6, and a maximum injection time of 200 ms. The samples were analysed in positive and negative mode in separate acquisitions.

Data for MS2 analysis were acquired in the Targeted SIM/dd-MS2 acquisition mode, with the following settings: (1) SIM with resolution of 70,000, an AGC target of 5e4, maximum injection time of 100 ms, loop count of 1, and isolation window of 1.0 m/z; (2) dd-MS2 with resolution of 35,000, an AGC target of 2e4, maximum injection time of 20 ms, loop count of 1, TopN of 1, isolation window of 2.0 m/z, and collision energy of 40 eV; (3) dd Settings: minimum AGC target of 8.00e2, intensity threshold of 4.0e4 and dynamic exclusion of 10 s. The samples were analysed in positive and negative mode in separate acquisitions. The inclusion list is given in Table S4.

### Metabolomics Data Analysis

Raw data files from the Q Exactive were converted into the mzML format by the ProteoWizard MS converter (Chambers et al., 2012). Data analysis was performed with the use of mzMatch, a modular, open-source, and platform-independent data processing pipeline for metabolomics LC-MS data written in the Java language implemented in R (Scheltema et al., 2011). Noise removal, signal filtering, and peak matching steps were performed. The detected features were grouped according to their likelihood to be associated with one single molecule, and only the most intense peak is considered for the subsequent statistical analysis. Putative annotation for the detected features was performed with the Integrated Probabilistic Annotation (Del Carratore et al., 2019, 2023), using an ad hoc database, including the known GE2270A congeners (Tocchetti et al., 2013). GE2270A was identified against the molecular weight and retention time of a standard. Processing and analysis of the fragmentation data was performed with the CluMSID R package (Depke et al., 2019).

### RNA Purification, QC, and Sequencing

Samples were collected at 21, 24, 28, 45, 48, 52, 68, 72, 76, and 92 hrs from a LW207 culture grown and at 15, 24, 39, 48, and 63 hrs from three *P. rosea* independent cultures in medium C and stabilised with 2 volumes of RNAProtect Bacteria reagent (Qiagen, Hilden, Germany) according to the manufacturer's instructions. Stabilised mycelia were kept frozen until processed. For RNA extraction, cell pellets were resuspended in 0.17 mL of lysozyme (15 mg/mL) and incubated at 30 °C for 10 min. Then, each suspension was transferred to a tube of lysing matrix B beads (MP Biomedicals, Loughborough, UK) containing 0.6 mL of RLT buffer (Qiagen, Hilden, Germany) supplemented with β-mercaptoethanol (100:1), and briefly vortexed. Total cell lysis was achieved by two pulses at 6.5 m/s, 30 s in a FastPrep instrument (MP Biomedicals, Loughborough, UK); samples were placed on ice between pulses. Centrifugation at room temperature and maximum velocity for 1 min served to compact the beads and recover the lysate. RNA was extracted with a mixture of acid phenol, chloroform, and isoamyl alcohol (25:24:1) in Phase Lock Gel tubes (5PRIME). Total RNA was purified according to the manufacturer's instructions with Direct-zol RNA MiniPrep Plus (Zymo Research, Irvine, California). The purity and concentration of RNA preparations were estimated using a NanoDrop 1000 (Thermo Scientific, Wilmington, Delaware). The integrity of RNA molecules was assessed through capillary electrophoresis with RNA Nano chips and a Bioanalyzer 2100 system (Agilent Technologies, Santa Clara, California). rRNA depletion, TruSeq library preparation, and RNA sequencing were conducted by vertis Biotechnologie AG (Freising, Germany). Briefly, rRNA molecules were depleted using an in-house developed protocol. Then, RNA samples were fragmented using ultrasound (4 pulses of 30 s each at 4 °C). After adapter ligation to the 3′ ends, first-strand cDNA synthesis was performed using Moloney murine leukemia virus reverse transcriptase and the 3′ adapter as a primer. After cDNA purification, the 5′ adapter was ligated to the 3′ end of the antisense cDNA. The resulting cDNA was amplified to about 10–20 ng/μL using a high-fidelity DNA polymerase and 15–16 PCR cycles. The cDNA was purified using the Agencourt AMPure XP kit (Beckman Coulter Genomics, Morrisville, North Carolina). Samples were pooled in approximately equimolar amounts and fractionated in a preparative agarose gel to recover molecules in the range of 180–600 bp. Since the total length of the primer sequences is 136 bases, RNA molecules approximately greater than 44 nucleotides were included in the cDNA libraries. Single-end sequencing was conducted on an Illumina NextSeq 500 system of 75-bp read length.

### RNAseq Bioinformatics Analysis

Fastq files containing the raw reads were processed with BBDuk and BBMap programs (B. Bushnell, sourceforge.net/projects/bbmap/). BBDuk served to remove adapter sequences (parameters: ′ktrim = r k = 23 mink = 11 hdist = 1′) and, in a second run, to filter reads by length and quality (′minlen = 20 maq = 10′). BBMap, run in local mode (′slow = t ambiguous = random′), served to map the filtered reads to the strain genome. The strain LW207 genome sequence and annotation were constructed from the GenBank entry NC_003888.3 (grandparent strain M145). Firstly, cosmid pbtCK02 sequence and annotation (accession KF366381.2) were inserted at the phiC31 *attB* site. Secondly, the four biosynthesis gene clusters deleted in parental strain M1146 were removed (those of actinorhodin, undecylprodigiosine, calcium-dependent antibiotic, and coelimycin) using the coordinates indicated by Gómez-Escribano & Bibb (2011). Reads mapped on both strands of the rRNA genes (corresponding to the annotated regions but increased 100 nucleotides upstream and 50 nucleotides downstream) were removed using the program split_bam.py of the RSeQC package (Wang et al., 2012). Final library sizes were in the range from 3.34 × 106 to 9.98 × 10^6^ reads. Alignment and genome data were processed in the R environment (version 3.6) using Bioconductor (version 3.9) (Gentleman et al., 2004) packages Rsamtools, GenomicFeatures, and GenomicAlignments (Lawrence et al., 2013). The summarizeOverlaps function of GenomicAlignments with mode ‘Union’ was used to count reads mapped to annotated genes. The transcript per million values (Wagner et al., 2012) were calculated from read counts using in-house spreadsheets.

### Proteomic Sample Preparation

For the preparation of *S. coelicolor* protein extracts from parental and derivative strains, all strains were cultured as described in Production of GE2270. Cell pellets were collected in biological triplicates at two different time points (24 and 96 hrs). For the preparation of *P. rosea* protein extracts, culture samples were collected at 24, 48, 72, 96, and 144 hrs from *P. rosea* grown in medium C. Cell pellets were harvested by centrifugation at 3000 rpm for 5 min at room temperature. The supernatant was carefully discarded, and the cell pellet was washed with sterile PBS twice. Cell pellets were gently resuspended in Urea Lysis Buffer (20 mM HEPES pH 8.0, 9 M urea, 1 mM sodium orthovanadate, 2.5 mM sodium pyrophosphate, 1 mM β-glycerophosphate) at 5:1 (vol: vol) to cell pellet volume. The cell lysate was mixed by gently pipetting, then using a microtip the cell lysate was sonicated at 15 W output, 3 × 15 s bursts cooling the lysate on ice between sonications. The sonicated lysate was centrifuged at high speed (14,000× *g*, 15 min at 4 °C), the supernatants containing the solubilised protein were transferred to a prechilled LoBind Eppendorf and stored at −20 °C to obtain the soluble protein extract.

### Untargeted Proteomic Sample Preparation

Solubilised protein extracts were digested using a combination of trypsin and Lys-C proteases, following reduction with dithiothreitol (DTT) and alkylation with iodoacetamide. The resulting peptides were desalted by C_18_ solid phase extraction (SPE) before loading onto LC-MS/MS. Peptides were eluted over a 2-hr acquisition from a 50 cm EN PepMap column driven by a Waters mClass UPLC onto an Orbitrap Fusion Tribrid mass spectrometer operated in data dependent acquisition (DDA) mode.

### Untargeted Proteomics Analysis

The resulting LC-MS chromatograms were loaded into Progenesis QI software for peak picking and alignment. A concatenated MS2 peak list was exported in .mgf format for database searching. Spectra were searched using Mascot against the *S. coelicolor* subset of UniProt appended with the Pbt proteins of *P. rosea* and common proteomic contaminants. Mascot results were combined and filtered using Percolator to require a peptide spectral match false discovery rate of <1%, as assessed against a reverse database search. Accepted search results were imported back into Progenesis QI and identifications associated with peptide MS1 precursors, identifications mapped between samples and the areas under the precursors integrated as a proxy for peptide abundance. Relative abundance was normalised between time points to total identified peptide ion areas. Protein abundance was estimated using the Top3 approach; summing the intensities for the three most intense peptide signals for each protein as a normalization for differences in peptide count due to protein length.

### Targeted PbtA Peptide Sample Preparation

Peptides were extracted from solubilised protein extract using C_18_ SPE. All samples were loaded under aqueous conditions and then eluted with 30% ACN. A 30% ACN elution was used so that longer proteins would be retained on the C_18_ clean-up and be less likely to interfere with subsequent LC-MS/MS. LC-MS/MS analysis was performed on the eluted peptides without protease digestion. Acquisitions were over 2 hr elution from a 50 cm EasyNano PepMap column driven by a Waters mClass UPLC onto a Thermo Orbitrap Fusion Tribrid mass spectrometer operated in DDA mode.

### Targeted PbtA Peptide Analysis

Combined data were searched using PEAKS Studio-XPro against the expected full-length sequence of PbtA (MSEMELNLNDLPMDVFEMADSGMEVESLTAGHGMPEVGASCNCVCGFCCSCSPSA). The search allowed for fragmentation at any amide bond and considered 313 of the most common post-translational modifications (PTMs). Peptide mass tolerance was restricted to 3 ppm and fragment mass tolerance to 0.5 Da. Matches were filtered in PEAKS to −log10P of >20, leaving the peptide matches as detailed in Fig. S3.

### Alphafold and AlphaPullDown

AlphaPullDown (v2.0.0)—a python package built around AlphaFold-Multimer (Yu et al., 2023)—was employed in ‘Pulldown’ mode to predict the structures of PbtX bound to other members in the GE2270A BGC. Multiple sequence alignments (MSAs) were created using MMseqs2 (Steinegger & Söding, 2017). All parameters were set to default except for the number of predictions per model (for a total of 50 models for each combination of PbtX bound to other members of the GE2270A BGC) and the inclusion of a relaxation step for all models. Predictions were assessed using 0.8 iPTM + 0.2 PTM scores (global quality score), pLDDT scores (local quality scores), and PI scores (Malhotra et al., 2021) (an independent measure of interface quality). We also produced individual structures for each protein using Colabfold (Mirdita et al., 2022) with default settings.

## Results and Discussion

### Generation of an M1146-Derivative With Improved Resistance to GE2270A

*Streptomyces coelicolor* M1146 has previously been demonstrated to exhibit antibacterial susceptibility to GE2270A, with detectable inhibitory effects on growth observed at amounts as low as 0.4 µg in an agar diffusion test (Flinspach et al., 2014). In the same study, attempts to enhance resistance to GE2270A by introducing the EF-Tu from *P. rosea* under the constitutive promoter ermE*p proved unsuccessful. We reproduced these results, confirming that this is not a feasible strategy for improving tolerance against our target compound (Fig. S1). It is possible that the translation machinery of *S. coelicolor* might largely disregard the heterologous elongation factor, albeit resistant, and use preferentially its cognate one. Based on this hypothesis, we explored two different strategies. First, we replaced the *tuf1* gene naturally occurring in the *S. coelicolor* M1146 genome with the resistant version from *P. rosea* using a CRISPR-Cas9 system (Cobb et al., 2015). While gaining resistance to at least 20 mg/L of GE2270A (Fig. S2), this approach severely impacted *S. coelicolor* growth, making the obtained strain unsuitable for future developments (Supplementary Data). Secondly, we engineered and synthetised a mutated version of the *S. coelicolor tuf1* gene in which 4-point mutations present in the *P. rosea* version, hypothesised to be involved in the GE2270A-resistant phenotype, were introduced. This mutated gene was used both for gene replacement using CRISPR-Cas9, and for ectopic integration (Supplementary Data). Both approaches proved unsuccessful. Unlike *P. rosea, S. coelicolor* has a second *tuf* gene (*tuf3*), which is expressed only under stress conditions (van Wezel et al., 1995), and codes for a protein that, albeit less active than EF-Tu1, is GE2270A-resistant (Olsthoorn-Tieleman et al., 2007). As described in the Supplementary Data, we built and tested three different constructs for the overexpression of *tuf3* in *S. coelicolor*, one under the control of the strong and constitutive ermE* promoter, one under the control of the anhydrotetracycline-inducible *tcp830* promoter, and one under the control of the thiostrepton-inducible *tipA* promoter. While the first two constructs did not confer any appreciable increase in resistance, the one induced with thiostrepton led to a resistant phenotype showing at least a fourfold enhanced resistance compared to the control strain (Fig. S4D). However, the obtained strain showed a drastic reduction of growth rate. Due to the slow growth and the dependence on induction with thiostreptone, we pivoted towards the isolation of spontaneous GE2270A-resistant mutants. To achieve this, we repeatedly subcultured *S. coelicolor* M1146 in media containing increasing sublethal concentrations of the thiopeptide antibiotic, a strategy that is frequently used to isolate spontaneous mutants with resistance to natural products (Fait et al., 2022; Nishimura et al., 2007). Following this approach, we isolated a derivative of *S. coelicolor* M1146 tolerating GE2270A up to 200 mg/L (Fig. S5). This strain, hereafter named LW200 (Fig. 1A), exhibited a significant improvement in GE2270A tolerance compared to *S. coelicolor* M1146, which showed a considerable level of growth inhibition at only 1.25 mg/L GE2270A (Fig. S5). Consequently, strain LW200 served as the basis strain for all further strain engineering aiming at improving the production of GE2270 in this host organism.

### Increasing the Copy Number of the Cluster-Associated Regulator Improves GE2270A Production

To investigate whether prior attempts to produce GE2270A in *S. coelicolor* were hampered by its sensitivity to the thiopeptide antibiotic, we integrated the native GE2270 BGC into strain LW200 at the phiC31 *attB* site using cosmid pbtCK02 (Flinspach et al., 2014). In the resulting strain, LW204, expression of the *pbt* genes comprising the GE2270 BGC is controlled by the native promoters from the cluster (Fig. 1B). Additionally, the integrated cosmid encodes *P. rosea* EF-Tu, which is constitutively expressed from an ermE* promoter (Flinspach et al., 2014). This strain yielded up to 1.42 ± 0.25 mg/L of GE2270A, a level comparable to what was obtained by Flinspach et al. (2014) by introducing an equivalent construct into *S. coelicolor* M1146 (i.e. the nonresistant parental strain of LW200).

As outlined in the introduction, the biosynthesis of GE2270A starts from the synthesis of a precursor peptide encoded by the *pbtA* gene. In *P. rosea*, the natural producer of GE2270A, *pbtA* is one of the top three most highly expressed genes in the whole genome (Del Carratore et al., 2021). We hypothesised that the bottleneck in the host organism might be the expression of *pbtA*. Therefore, we inserted an additional copy of the gene encoding the precursor peptide under the control of the *act1* promoter. The strong SP44 promoter (Bai et al., 2015) was also tested, showing no significant changes in GE2270A titres. This strategy did not yield the desired results; in fact, the obtained strain, named LW205 (Fig. 1B), produced 1 ± 0.3 mg/L of GE2270A, with no statistically significant difference when compared with the yield obtained from LW204.

The overexpression of transcriptional activators that positively regulate the expression of BGCs of interest is often a successful strategy for yield increase (Xia et al., 2020; Yamanaka et al., 2020). It has been shown that PbtR, the cluster-associated transcriptional regulator, plays an essential role in the biosynthesis of GE2270A and its deletion results in the complete abolishment of GE2270A production (Flinspach et al., 2014). Following this rationale, we inserted an additional copy of *pbtR* under the control of the kasO* promoter, which resulted in the LW207 strain (Fig. 1B). This strain was able to produce up to 3.44 ± 1.07 mg/L of the target compound, resulting in a 2–3× increase compared to LW205 and LW204.

With the goal of finding genetic targets for further yield improvement, LW207 was subjected to a multiomics analysis where we acquired untargeted metabolomics, transcriptomics, and proteomics data. All data acquired from this experiment can be found in the Supplementary Data. Untargeted metabolomics and transcriptomics data obtained following *P. rosea* fermentation was already publicly available (Del Carratore et al., 2021). In this study, we also acquired proteomics data for the GE2270A natural producer, also available in the Supplementary Data, which allowed for an extensive comparison between strain LW207 and *P. rosea*.

From the proteomics data obtained from the fermentation of *P. rosea*, we were able to identify the complete PbtA precursor peptide. As shown in Fig. S7, the proteomics data indicate that the correct translational start site of *pbtA* is downstream of the one that was annotated in the published genome. This is also supported by the presence of a strong Shine-Dalgarno sequence present 8 bp upstream of the correct start codon (AGGAGA).

The analysis of the already published transcriptomics data of *P. rosea* revealed that almost all ribosomal genes followed the same trend, suggesting a strong reduction in protein biosynthesis after 24 hrs (Del Carratore et al., 2021). The only exception is a gene annotated as ‘30S ribosomal protein S4’ the expression of which peaked at 39 hrs, suggesting that this protein might be involved in the target compound's biosynthesis (Del Carratore et al., 2021). Starting from the LW204 strain, we therefore inserted an additional copy of the correct version of the gene encoding the precursor peptide, hereafter named *pbtA_full_*, and a copy of the gene *3163S4* (i.e. the gene annotated as ‘30S ribosomal protein S4’ in *P. rosea*) under the control of the *ermEp1* promoter (Bibb et al., 1985). Through this strategy, we obtained the strain named LW292 (Fig. 1B), which yielded 1.39 ± 0.83 mg/L of the target compound, hence not providing any improvement compared to its parental strain LW205.

Similarly to what was done for LW207, we obtained strain LW296 by inserting an additional copy of *pbtR* into LW292, also under the control of the *ermEp1* promoter. This strain produced 4.8 ± 1.6 mg/L of the target compound, representing a 3× yield increase when compared to LW292 and slightly better than strain LW207, confirming the positive effect of an additional copy of *pbtR*.

The comparison of metabolomics data obtained from *P. rosea* and LW207 (Fig. S6) showed that LW207 produced a higher number of linear congeners (relative to the final product). All the known congeners, the structures of which have been characterised, are summarised in Fig. S9. We therefore hypothesised that cyclisation might be a bottleneck. As a result, we inserted an extra copy of *pbtD* into the LW296 strain, resulting in the LW298 strain (Fig. 1B). This approach did not produce the desired results; in fact, LW298 yielded 3.63 ± 1.7 mg/L of the targeted compound, which is, although not statistically significant, lower than what was obtained from LW296.

### Comparing Gene Expression and Protein Levels of the GE2270-related Genes Between Natural Producer and the Heterologous Host

As highlighted in Fig. 1A, metabolomics, transcriptomics, and proteomics data were acquired for a subset of the strains generated in this study. Additionally, proteomics data was acquired for *P. rosea* in addition to the metabolomics and transcriptomics data previously acquired and already published (Del Carratore et al., 2021). All data are available in the Supplementary Data.

**Fig. 2.**
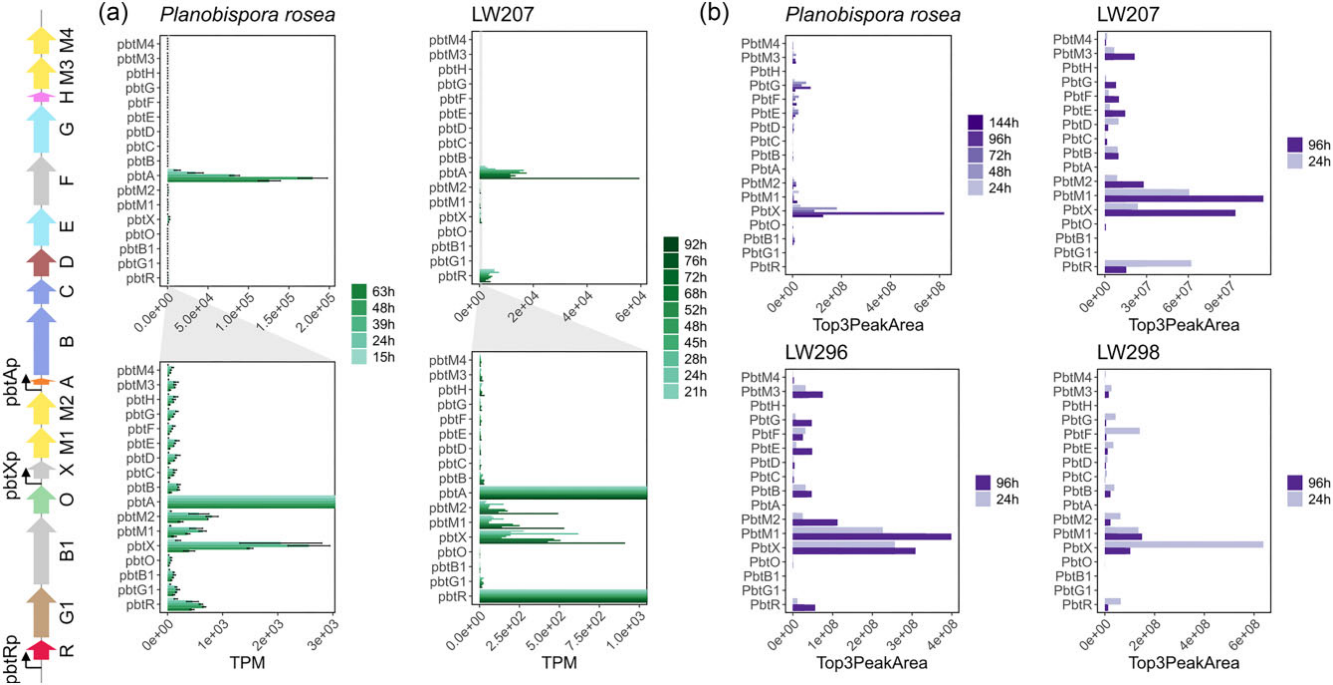
Gene expression and protein levels of the GE2270 cluster for a selection of strains considered in this study. (A) Gene expression levels of the genes from the GE2270 BGC measured in the natural producer *P. rosea* (left) and strain LW207 (right) at different time points. Gene expression levels are shown in transcripts per million (TPM). (B) Protein levels measured for the GE2270 BGC in the natural producer *P. rosea* and three *S. coelicolor* strains: LW207, LW296, and LW298.

The transcriptomics data allowed for a direct comparison between the gene expression levels observed in the natural producer (*P. rosea*) and in the heterologous host (*S. coelicolo*r LW207). As shown in Fig. 2A, the most highly expressed gene of the *pbt* cluster is *pbtA*, which is observed in both the natural and heterologous host. In LW207, the second most expressed gene is *pbtR*, which is likely due to the additional copy integrated in this strain (see Fig. 1B). In *P. rosea, pbtR* is not as highly expressed, while the operon comprising *pbtX, pbtM1* and *pbtM2* appears to be highly expressed when compared to the rest of the cluster. The same can also be observed in LW207 and among these three genes *pbtX* is the most highly expressed in both strains. Fig. 2B shows the protein levels detected in *P. rosea* and in three *S. coelicolor* strains (LW207, LW296, and LW298). In a previous experiment, we were able to detect the precursor peptide, which led to the identification of the true translational start site of PbtA (Fig. S7). In the subsequent analysis of the whole proteome of *P. rosea*, we were able to detect 15 out of the 17 proteins encoded within the *pbt* cluster at varying levels across all different time points, while both PbtA and PbtH were not detected. Consistently with the transcriptomics data, the level of PbtX is the highest at all time points, with maximum abundance detected at 96 hrs correlating with the highest production of GE2270A in *P. rosea*. PbtR is close to the detection limit and its level appears to decrease at the later time points. In the *S. coelicolor* strains, we were able to detect 13 out of the 17 *pbt* cluster proteins at levels that are comparable to what was observed in *P. rosea*. PbtO, however, is only matched by a single peptide, making the result less confident for this protein. In this case, PbtA and PbtH were also not detected. Interestingly, we also did not detect PbtG1 or PbtB1, suggesting that they might be responsible for the bottleneck in the GE2270A biosynthesis in the heterologous host, as they are both core proteins of the *pbt* cluster. In all *S. coelicolor* strains, PbtX and PbtM1 were consistently the proteins detected at highest levels. The gene *pbtX* is located within the cluster; to date, there has been no proposed role for this protein. In *P. rosea*, PbtX is expressed in a GE2270A-dependent manner, suggesting it may have an important role in GE2270A production.

It should be noted that Tocchetti *et al*. (2013) previously obtained a *Nonomuraea* ATCC39727 strain expressing the *pbt* cluster lacking the *pbtX* gene. This strain showed no production of GE2270A, and the two major compounds detected were the congeners B1 and D1. Very similar results were found in the strain lacking *pbtM1*. The authors could not exclude polar effects of the deletion of *pbtX* on the expression of *pbtM1*. The data collected here strongly suggest that *pbtX* plays an important role in GE2270A biosynthesis, possibly in combination with *pbtM1*.

To further investigate this hypothesis, we employed the AlphaPullDown pipeline (v2.0.0) (Yu et al., 2023) to assess *in silico* the likelihood of PbtX forming a protein complex with any of the other proteins present in the GE2270A BGC. As shown in Fig. S8, PbtF is the only protein of the GE2270A cluster that is likely to bind with PbtX. Interestingly, PbtF has been recently shown to bind to an 8-residue fragment of the precursor peptide PbtA (unpublished, https://www.rcsb.org/structure/8t19), suggesting a possible interaction between these two proteins. However, it should be noted that AlphaFold (Abramson et al., 2024; Jumper et al., 2021) shows a relatively low confidence in the predicted structure for PbtX (also reflected in the local quality scores for PbtX in complex with PbtF), which increases the uncertainty around the protein's binding predictions.

### Cluster Refactoring By Introducing Constitutive Promoters and Generation of a Minimal Cluster Also Yield No GE2270A

**Fig. 3.**
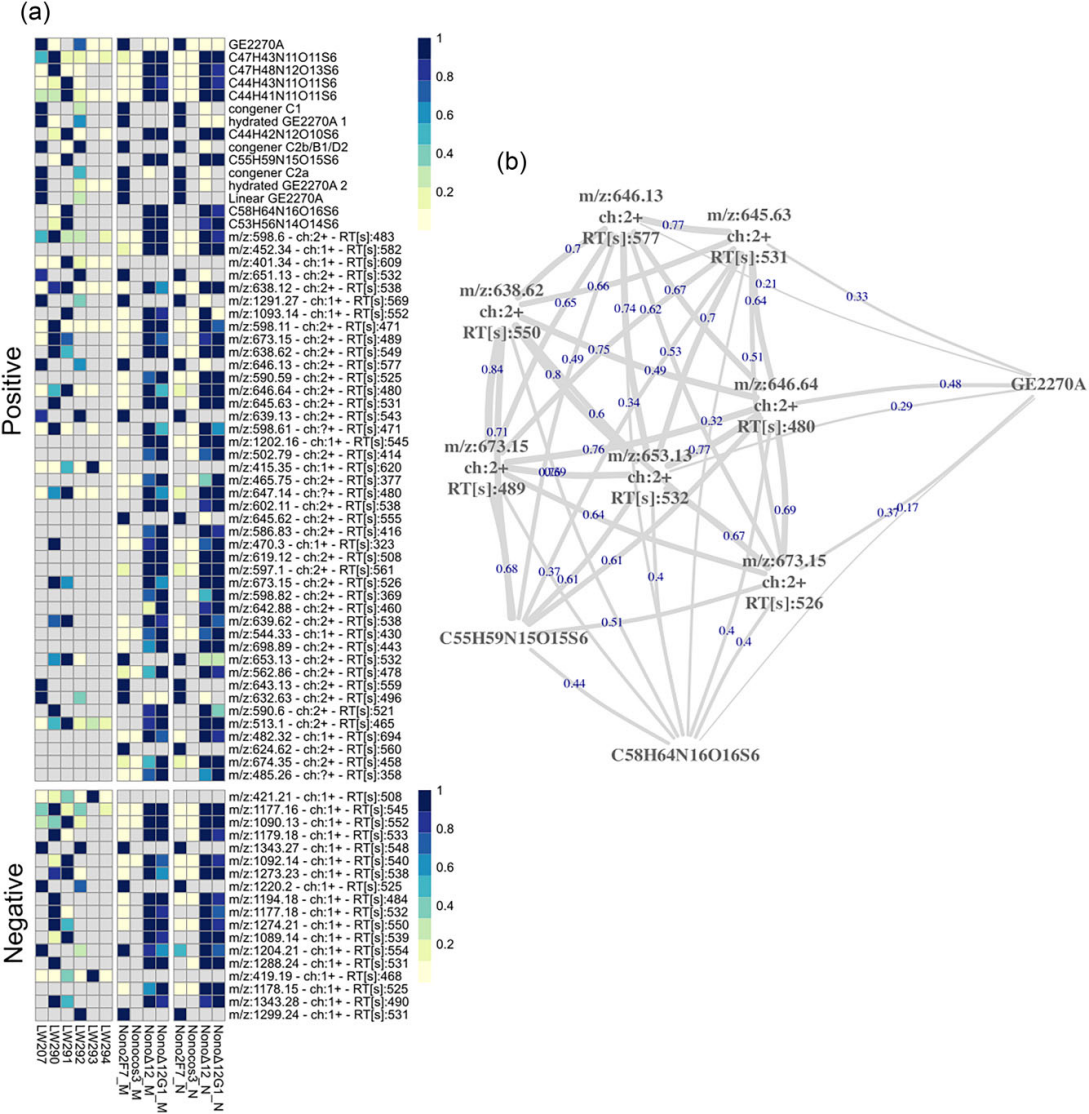
Summary of the GE2270-associated peaks detected in an untargeted metabolomics experiment considering whole-broth extraction of the fermentation of six *S. coelicolor* strains (LW207, LW290, LW291, LW292, LW293, and LW294) and four *Nonomuraea* strains (Nono2F7, Nonocos3, Nono-Δ12, and Nono-Δ12pbtG1). The *S. coelicolor* strains were analysed at the University of Manchester, while the *Nonomuraea* strains were analysed both in MEDINA (M) and Naicons (N) labs. (A) Heatmap showing the relative abundance (normalised by row) of the GE2270-related peaks detected in positive and negative mode. The features without any annotation mass-to-charge ratio (m/z), predicted charge and retention time are shown. (B) Similarity network for the fragmentation spectra acquired for ten of the selected features related to GE2270A from the *S. coelicolor* strains. All features considered here show a significant similarity in the fragmentation spectra with GE2270A, hence providing additional evidence that these features are indeed related with the GE2270 cluster.

Since the introduction of additional copies of cluster-associated genes, as well as the gene encoding the ribosomal protein S4, had only modest effects on GE2270 biosynthesis, we sought to uncouple the expression of the genes within the *pbt* cluster from their native regulation. This was achieved by replacing the native promoters with strong constitutive promoters. Initially, we eliminated the operon encoding PbtRG1B1O and replaced the native *pbtX* and *pbtA* promoters with the strong constitutive A9p and 21p (Siegl et al., 2013), respectively (Fig. 1). In the resulting strain, LW290, the production of GE2270A and its congeners was completely abolished. Notably, even though the deletion of *pbtG1* in *Nonomuraea* ATCC 39727 carrying the 2F7 cosmid has been previously shown to yield linear precursor molecules (Tocchetti et al., 2013), linear GE2270A could not be detected in LW290 either (Fig. 3A). Consequently, *pbtG1* was reintroduced under the control of an *ermEp1* promoter, yielding strain LW291 (Fig. 1). In this strain, *S. coelicor*’s ability to form cyclised congeners of GE2270 was restored (Fig. 3A).

Simultaneously, we generated a minimal *pbt* cluster that lacks all the enzymes responsible for methylation and hydroxylation modifications. The resulting strain, LW293, was unable to produce GE2270A. This was also the case for strain LW294, which included all the genes of the *pbt* cluster, but in an altered gene order (Fig. 1B). It was only when all the genes of the *pbt* cluster were assembled in their original gene order, resulting in strain LW295, and despite being expressed from constitutive promoters, that we could restore the production of GE2270A, albeit at levels considerably lower than in LW204, which harbours the unmodified GE2270 BGC. These data suggest that the natural operon structure is important for the synthesis of a functional pathway for GE2270 biosynthesis. Combined with our data suggesting that certain genes such as *pbtX* are highly expressed in the natural cluster, yet are of unknown function, this indicates that there is more complexity in the natural operon function and regulation than perhaps first assumed.

### *Nonomuraea* as Alternative Expression Host

Tocchetti et al. (2013) demonstrated that *Nonomuraea* ATCC 39727 is a good host for the heterologous expression of the *pbt* cluster from *P. rosea*. The resulting strain, hereafter called Nono-2F7, was able to produce up to 250 mg/L of GE2270A when grown in C1 medium. Regardless of our best efforts, the rational refactoring of the *pbt* cluster in *S. coelicolor* resulted in only minor yield improvements. In fact, the highest production level observed in *S. coelicolor* is 12× lower than what was observed in the natural producer and 50× lower than what was published using *Nonomuraea* ATCC 39727, as expression host for a nonrefactored *pbt* cluster, when cultivated under optimised conditions. With the aim of exploiting the superior production capability of this *Nonomuraea* strain to better understand the biosynthetic activities encoded by the truncated *pbt* clusters, the pTE1710 and pTE1711 plasmids (Table S3) were transferred by conjugation into *Nonomuraea* ATCC 39727 yielding the strains Nono-Δ12 and Nono-Δ12pbtG1. Nono-Δ12 therefore contains the *pbt* cluster lacking *pbtR, pbtG1, pbtB1*, and *pbtO*, similarly to *S. coelicolor* strain LW290. Nono-Δ12pbtG1 contains the same plasmid where *pbtG1* was reintroduced, similarly to *S. coelicolor* strain LW291. No production of GE2270A was detected in these strains. To identify the compounds produced by these truncated clusters, the metabolite profiles of these strains were compared with a selection of the *S. coelicolor* strains constructed in this study and the Nono-cos3 strain carrying the empty vector. This was done through an untargeted metabolomics analysis of the whole-broth acetonitrile extracts of samples collected from the fermentation of the strains considered. By comparing the metabolic profiles obtained with those detected in the strain lacking the *pbt* cluster vector (i.e. not able to produce any GE2270-related compounds), we identified several mass-spectrometry features associated with the *pbt* cluster as shown in Fig. 3A. It should be noted that the results obtained from the *Nonomuraea* strains are extremely reproducible, despite being analysed in two different institutes, and the diversity and relative abundance of the various congeners is much higher than in the corresponding *S. coelicolor* strains. For a selection of 10 of these features, including the main product GE2270A, fragmentation data was acquired. As shown in Fig. 3B, all spectra acquired show high similarity, hence providing additional evidence that these are indeed features related to the *pbt* cluster. From the fragmentation data, two previously unreported compounds were identified as shown in Fig. S9, which had not been seen in *S. coelicolor*. Revisiting the raw MS data confirmed that these compounds are also produced in *S. coelicolor* LW290 and LW291, but at levels that are too low for confident detection and insufficient for MS/MS fragmentation. This confirms the value of *Nonomuraea* as an alternative host, not only for the production of GE2270A, but also as a platform organism for elucidating the molecular details of the biosynthetic pathway.

## Conclusion

In this study, we aimed to enhance the production of the clinically relevant RiPP GE2270A through heterologous expression of its BGC in *S. coelicolor* M1146, which is a valuable model organism for investigating the genetic regulation of antibiotic production in *Streptomyces*. Through a data-driven rational cluster engineering approach, involving the introduction of additional copies of crucial genes, we were able to obtain a statistically significant increase in the GE2270A yield. Despite our efforts, however, the highest production level observed remained significantly lower than what was published for the natural producer (*P. rosea*) or using the *Nonomuraea* ATCC 39727 as expression host. Moreover, the analysis of the multiomics data obtained in this study highlighted the potential importance of PbtX in GE2270A biosynthesis and provided valuable insights into the differences observed in the metabolic profiles of the different strains investigated in this study. This study serves as a cautionary tale for scientists interested in engineering and refactoring BGCs for heterologous production of natural products. *Streptomyces coelicolor* was selected as host organism due to its genetic tractability and a wealth of tools available for its genetic engineering; however, despite these advantages, it proved not to be the ideal host to produce GE2270A. This highlights the importance of careful host selection, considering additional factors such as resistance to the target compound, availability of genetic tools, and the potential for high yields. In the case of GE2270A, the alternative host, *Nonomuraea* ATCC 39727, proved to be a superior host able to achieve much higher yields. Additionally, the study emphasises the complexity of natural product biosynthesis and the need to consider intricate regulatory mechanisms in both the host and the BGC, that are not always known and may prevent the achievement of substantial yield improvements, even with rational engineering approaches.

## Supplementary Material

kuaf019_Supplemental_Files

## Data Availability

The data underlying this article are available in the article and in its online supplementary material, as well as in the associated GitHub repository https://github.com/francescodc87/Engineering-Streptomyces-coelicolor–a-cautionary-tale.
