# Supplementary material for: Engineering Streptomyces coelicolor for heterologous expression of the thiopeptide GE2270A—A cautionary tale
Source: J Ind Microbiol Biotechnol. 2025 Jul 18;52:kuaf019. doi: 10.1093/jimb/kuaf019 (PMC12309360; doi:10.1093/jimb/kuaf019)
Supplement: kuaf019_Supplemental_Files [file kuaf019_supplemental_files.zip › Supplementary_material_cautionarytale_clean.docx]

Engineering *Streptomyces coelicolor* for heterologous expression of the thiopeptide GE2270A – a cautionary tale

# Supplementary material

**Attempts to improve GE2270A tolerance of S. coelicolor thorugh the overexpression of different resistant versions of EF-Tu protein**

The first GE2270A heterologous producer, *S. coelicolor* M1146::pbtCK02 (LW221) is still sensitive to GE2270A (Flinspach et al., 2014) despite possessing the resistant gene, Pr*tuf* (*tuf* gene from the natural producer *Planopbisporaea rosea*), under the control of the constitutive promoter, *ermE**p. We reproduced these results during the optimisation of the GE2270 production conditions, when agar diffusion bioassays of cell extracts only showed a very slight improvement, if any, in GE2270A tolerance relative to the parental strain (**Fig. S1**). This very low level of tolerance is not likely to protect any future GE2270A-overproducer strain, and we set out to find ways to increase it.

**Figure S1. Agar diffusion bioassays of GE2270A tolerance by M1146 and M1146::pbtCK02.** Numbers indicate amounts of pure GE2270A in μg. The arrow depicts the minimal amount of GE2270A in which bioactivity is detected.

To increase GE2270A tolerance by M1146, the overexpression of different resistant versions of the EF-Tu protein were attempted: *P. rosea* tuf (Pr*tuf*); a mutated version (muttuf1) of *S. coelicolor*’s *tuf*1 containing 4 point mutations present in Pr*tuf* (G257S, V274A, G275A, V291C); and a secondary EF-Tu coding gene *tuf*3 present in *S. coelicolor*’s genome (Sc*tuf*3).

Over-expression of *Planobispora rosea tuf* (Pr*tuf*)

As discussed above, over-expression of Pr*tuf* using the strong *ermE**p promoter has already been attempted to negligible effects^1^ (**Fig S1**). We reasoned that, when both *P. rosea* and *S. coelicolor* EF-Tu are present, the translation machinery of *S. coelicolor* might largely disregard the heterologous, albeit resistant version, and use preferentially its cognate, though sensitive EF-Tu. This would indeed result in cells that are still sensitive to GE2270A. Based on this hypothesis, one of the options we have explored was to replace the Sc*tuf*1 on the M1146 chromosome with Pr*tuf*, in an attempt to force the strain to use the resistant variant. To do so, we decided to implement the CRISPR-Cas9 system^2^, obtaining strain LW257. As shown in **Fig. S2**, LW257 resistance against GE2270A was tested by plating it on SFM + GE2270A (20 mg/L). Parental strain LW221 was also grew as control.

**Figure S2. Bioassay testing resistance to GE2270A of strains LW221 and LW257.**

While this approach was successful in conferring resistance, it severely impacted *S. coelicolor* growth, making the obtained strain unsuitable for future developments.

Expression of a mutated version of *Streptomyces coelicolor tuf*1

Another way to engineer resistance via EF-Tu is to modify *S. coelicolor* EF-Tu amino acid sequence to that of *P. rosea*. Therefore, a mutated version of the Sc*tuf*1 was synthesised, in which 4 point mutations were introduced (G257S, V274A, G275A, V291C), all of them present in Pr*tuf* and thought to be involved in its GE2270A-resistant phenotype and not present in *S. coelicolor*’s *tuf1*. This synthetic construct was ordered as a 2 kb length gBlock to IDT Technologies (Ref. 74028784) and cloned into pCR-Blunt, to generate the construct pBluntmuttuf1. This mutated gene was used both for gene replacement using CRISPR-Cas9 and for ectopic integration at a different site on the genome. For ectopic integration, pIJ1025Amuttuf1 construct was generated, which is a BT1 integrative vector with mut*tuf*1 under the control of the natural Sc*tuf*1 promoter. This construct was introduced into M1146 by intergeneric conjugation into the phiC31 integration site, generating the strain M1146::pIJ10257Amut*tuf*1. Two independent colonies, M1146::pIJ10257Amut*tuf*1 (1) and (3), were tested for their resistance to GE2270A, which was measured by agar diffusion bioassays using *Bacillus subtilis* 168 as indicator strain. Known concentrations of pure GE2270A were inoculated into wells made in the agar, and the plates were incubated for 48h at 30°C. M1146 was used as a control. No differences were observed between the strains (**Fig. S3**), which may be due to the mutant EF-Tu being a poor functional replacement for WT EF-Tu.


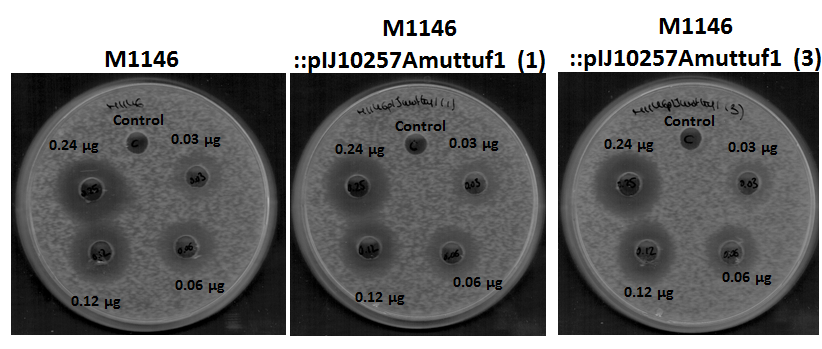


**Figure S3. GE2270A tolerance testing of a refactored strains expressing a mutant allele of Sc*tuf*1 as indicated above panels.** Numbers denote given concentrations of pure GE2270A.

Over-expression of *Streptomyces coelicolor tuf*3

Unlike *P. rosea*, which only has one tuf gene, many Streptomycetes present two or even three different *tuf* genes. In fact, *S. coelicolor* possess two: Sc*tuf*1, coding for the house-keeping EF-Tu, which is GE2270A-sensitive and has been discussed above, and Sc*tuf*3, which is expressed only under stress conditions (e.g., amino acid starvation)^3^, and codes for a protein that, albeit less active than EF-Tu1, is instead GE2270A-resistant^4^.

We overexpressed Sc*tuf*3 , where an overexpressed plasmid-borne copy of Sc*tuf*3 did confer GE2270A resistance to *S. coelicolor*. To test this, the Sc*tuf*3 was PCR amplified using *S. coelicolor* M145 (M1146 parental strain) genomic DNA as template, followed by cloning into pCR-Blunt to give pBlunttuf3, which was confirmed by sequencing. Three different expression constructs of Sctuf3 were constructed:

- pIJ10257A*tuf*3: here Sc*tuf*3 is placed under the control of the strong and constitutive *ermE** promoter in an integrative vector that uses phage BT1 integrase.
- pAV11bA*tu*f3: here Sc*tuf*3 is placed under the control of the aTc-inducible tcp830 promoter in an integrative vector that uses phage BT1 integrase.
- pIJ8781*tuf*3: this is a self-replicative construct where Sc*tuf*3 is placed under the control of the thiostrepton-inducible tipA promoter in a multiple copy plasmid.

Each of the constructs were introduced into M1146 by intergeneric conjugation generating the corresponding M1146::pIJ10257ASc*tuf*3, M1146::pAV11bASc*tuf*3 and M1146 (pIJ8781ASc*tuf*3) strains. A negative control strain, M1146::pIJ10257A, was also created during the same procedure.

Two independent clones per genotype were tested by agar diffusion bioassays using *Bacillus subtilis* 168 as indicator strain; when required, inducers, i.e., aTc (for pAV11b) and thiostrepton (for pIJ8781) were added to the medium. None of the strains carrying an ectopic integrated copy of Sc*tuf*3 displayed any increase in resistance either relative to their empty vector strain (M1146::pIJ10257*tuf*3, **Fig. S4A**) or in the absence of induction (M1146::pAV11b*tuf*3, **Fig. S4B**). However, the plasmid-borne version of Sc*tuf*3 (M1146::pIJ8781*tuf*3), induced with thiostrepton, did lead to a resistant phenotype showing at least 4x fold resistance relative to untransformed M1146 (**Fig. S4D**), although the growth of the plasmid-bearing strains was severely affected (**Fig. S4C**).


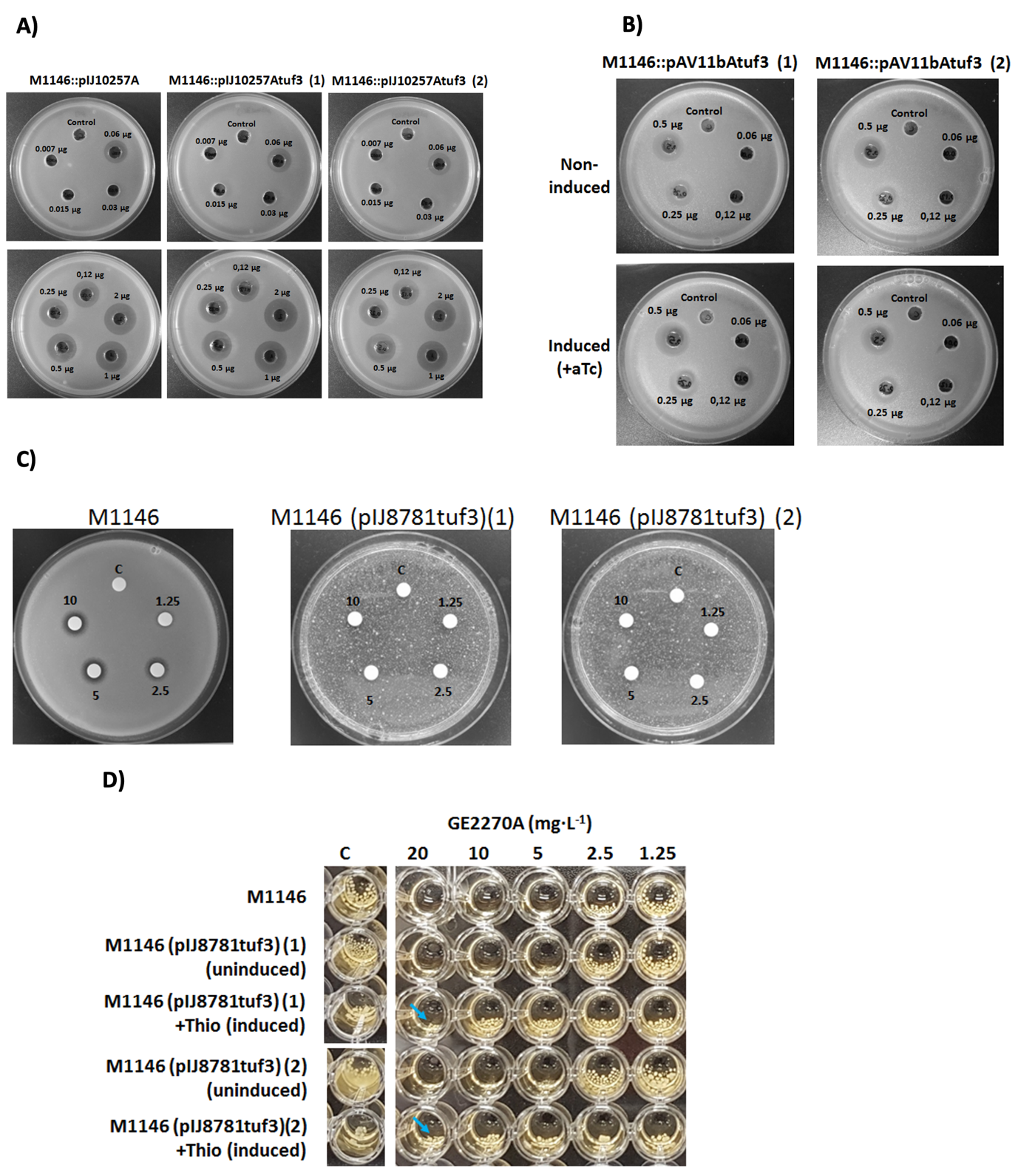


**Figure S4. GE2270A tolerance of refactored strains expressing Sc*tuf*3 by agar diffusion assays with *B. subtilis* as indicator strain and by using known amounts of GE2270A.** A) M1146::pIJ10257A*tuf*3 (two clones and M1146::pIJ10257A as control strain) B) M::pAV11bA*tuf*3 (two clones uninduced and induced conditions) and C) M1146 (pIJ8781*tuf*3) (two clones and parental strain as control). D) Liquid culture assays testing M1146 (pIJ8781*tuf*3) in a 96-well plate by using known concentrations of GE2270A.

To investigate the phenotype of M1146 (pIJ8781*tuf*3) further, we performed liquid bioassays (in this case, growth needed to be incubated for 72h instead of 48h, due to the slower growth rate of the strains). Both clones of M1146 (pIJ8781*tuf*3) showed at least 4 fold increased resistance to GE2270A when the expression of Sc*tuf*3 was induced with thiostrepton, being able to grow even at 20 mg/L of GE2270A, which was the highest concentration tested (**Fig. S4D**). Amongst all the different recombinant strains generated in order to increase their tolerance to GE2270A, only M1146::pIJ8781*tuf*3 showed positive results, displaying an increased MIC of at least 4 fold in comparison to the parental strain. This is consistent with the hypothesis that GE2270A-resistant forms of EF-Tu need to be very highly expressed in order to functionally replace the house-keeping EF-Tu1. However, this is not an optimal strain, because of its slow growth and its dependence on induction (thiostrepton). This prompted us to seek other ways for improving intrinsic resistance, including the isolation of spontaneous GE2270A-resistant mutants.

**Isolation of *S. coelicolor* M1146 spontaneous mutants resistant to GE2270A**

*Streptomyces coelico*r M1146 was repeatedly subcultured in media containing increasing sublethal concentrations of GE2270A.


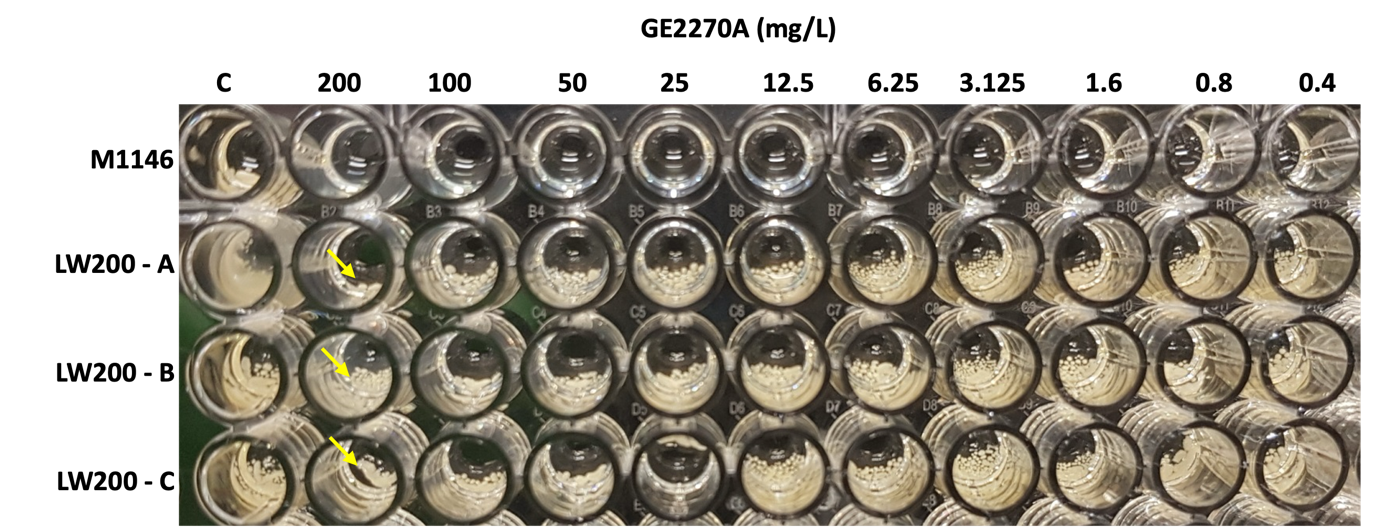


**Figure S5. GE2270A tolerance of spontaneous mutant strain LW200.** Liquid bioassays of three biological replicates of strain LW200 (A–C) and the parental strain M1146. Strains were grown in a 96-well microtitre plate in the presence of a wide range of GE2270A concentrations.

**Comparison of relative levels of GE2270 congeners produced by different strains**

Using the previously published untargeted metabolomics data for *Planobispora rosea*^5^ and untargeted metabolomics data acquired in this study, we compared the relative levels of congeners produced by the natural producer and the LW207 strain.


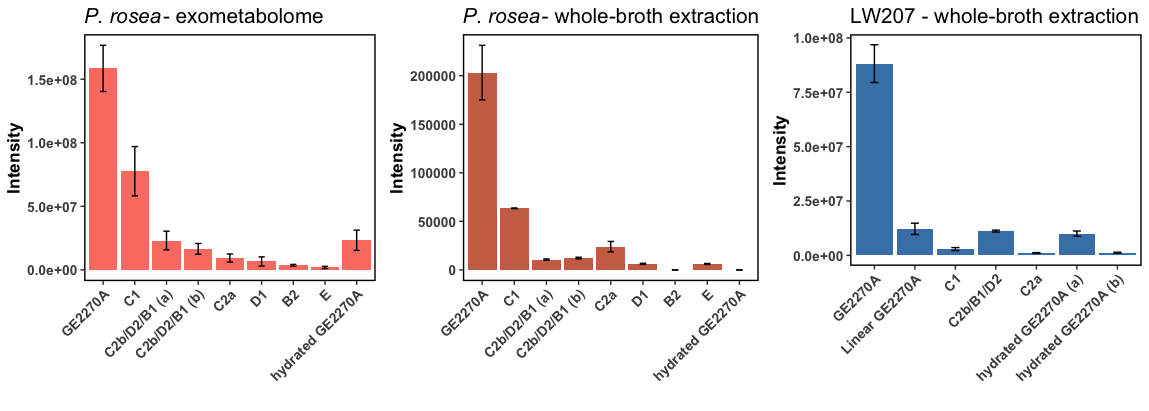


**Figure S6. Comparison of the GE2270 congeners produced by *P. rosea* and LW207.** The figure shows the relative intensities associated with the culture of the two different strains at the latest timepoint, when the main congener concentration (GE2270A) is the highest. The linear congener is only detected in the LW207 strain.

**Identification of true translational start site of PbtA through proteomics**

The proteomics analysis allowed the detection of the leader peptide as well as the identification of the true translational start site of PbtA.

#
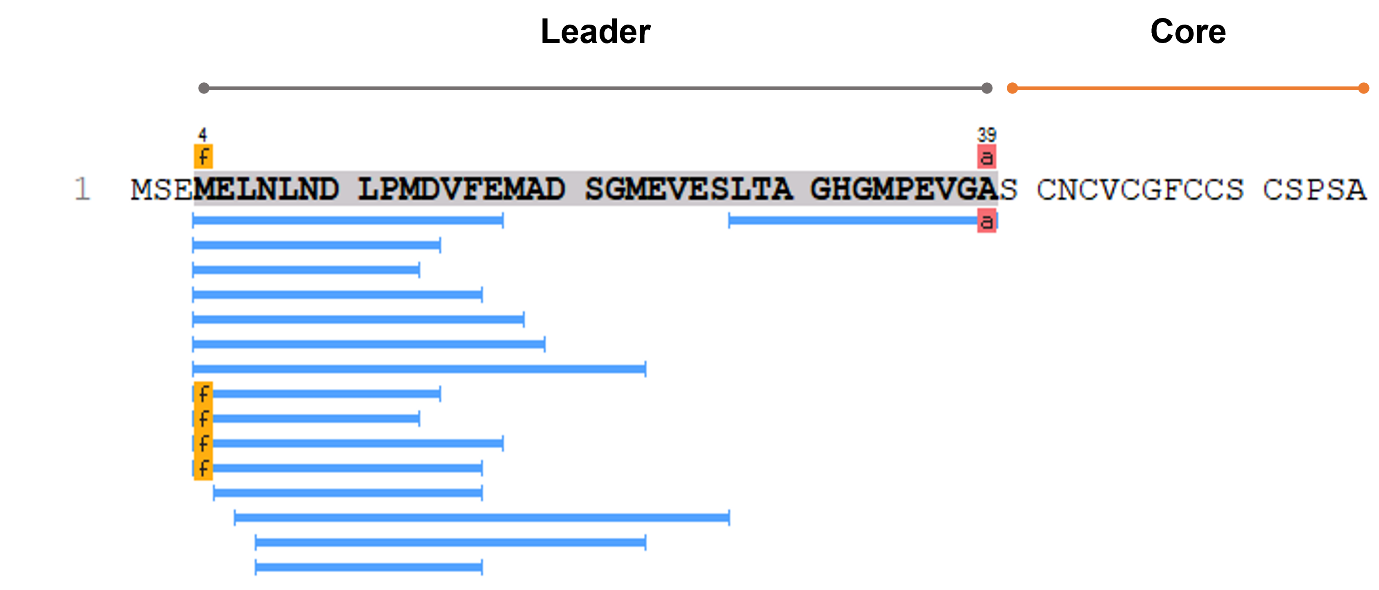


**Figure S7. Detection of PbtA peptide fragments.** Detection of PbtA fragments in *P. rosea*. Multiple peptide forms are seen towards the N-terminal side. The coverage abruptly stops at the active thiopeptide, which itself is not matched. It may be that the peptide is here in the active cyclic form and not being matched for that reason. The LC-MS/MS approach is not ideal for cyclic peptides as fragmentation tends to open the ring rather than fragment the peptide, resulting in no change in mass and little information in the MS2 spectrum.

**Supplementary Table S1**. Strains used and generated in this study.

| Strain | Genotype/characteristic | Reference or source |
| --- | --- | --- |
| *E. coli* |  |  |
| ET12567/pUB307 | Conjugation donor | MacNeil *et al.*, 1992^6^  Bennet *et al.*, 1977^7^ |
| ET12567/pUZ8002 | Conjugation donor | MacNeil *et al.*, 1992^6^ |
| *E. coli* NEB5α | *fhuA2Δ(argF-lacZ)U169 phoA glnV44 Φ80Δ(lacZ)M15 gyrA96 recA1 relA1 endA1 thi-1 hsdR17* | NEB |
| *S. coelicolor* |  |  |
| M1146 | Δ*act* Δ*red* Δ*cpk* Δ*cda* | Gomez-Escribano *et al.*, 2011^8^ |
| LW200 | M1146, spontaneous mutant tolerant GE2270A | This study |
| LW204 | LW200::pbtCK02 | This study |
| LW205 | LW200::pbtCK02::pES118 | This study |
| LW207 | LW200::pbtCK02::pES118:: pES94* | This study |
| LW290 | LW200::pTE1710 | This study |
| LW291 | LW200::pTE1711 | This study |
| LW292 | LW204::pTE1719 | This study |
| LW293 | LW200::pTE1712 | This study |
| LW294 | LW200::pTE1713 | This study |
| LW295 | Derivative of LW204; P*_ermE1_*-*pbtRG1B1O*, P*_A9_*-*pbtXM1M2*, P*_21_*-*pbtABCDEFGHM3M4* | This study |
| LW296 | LW204::pTE1720 | This study |
| LW298 | LW204::pTE1721 | This study |
| *N. gerenzanensis* |  |  |
| Nono-2F7 | Nonomuraea::2F7 | Flinspach *et al.*, 2014^1^ |
| Nono-cos3 | Nonomuraea::SuperCos3 | Flinspach *et al.*, 2014^1^ |
| Nono-Δ12 | Nono::pTE1710 | This work |
| Nono-Δ12pbtG1 | Nono::pTE1711 | This work |

**Supplementary Table S2.** Oligonucleotide primers used in this study.

| **Oligonucleotide primer name** | **Sequence (5′ to 3′)** |
| --- | --- |
| 3163_S4-pRT-f | tagcgggcagggagcggttgtgggctggacaatcgtgccggttggtaggatccagcgggtatgctaaggaggcaacaagtgcgctacaccggacc |
| 3163_S4-pRT-r | acagctatgacatgattacgaattcgattcagcgggagtagaactccac |
| 3163-UNS6-SspI-f | cgctgccacctaagaatattaatactctacggtcacatactgctaaggaggcaacaagtg |
| 3163pbtAf-pRT-r | cacgggatcgcgaagactgttcagcgggagtagaactcca |
| A9_pbt02X-ps-r | gtatgtgaccgtagagtattcttaggtggcagcgaacgagcagaccccgtagaaaaga |
| A9_pbt02X-1.2-f | caagacgctggctctgacatttccgctactgaactactcgacgctcagtggaacgaaa |
| A9-pbt02X-fF-f | ctcgttcgctgccacctaagaatactctacggtcacataccgccgctggcgcggctgctc |
| A9_pbt02X-fF-r | ccctttatcctagtcaagtcaagcatgaggaagccgcccggaaaaccgtattctcgactt |
| A9_pbt02X-fR-f | ccgggcggcttcctcatgcttgacttgactaggataaaggggaatcagcgagcagaaaga |
| A9_pbt02X-fR-r | cgagtagttcagtagcggaaatgtcagagccagcgtcttggatgctcgggcaggccgtcg |
| A9_pbt02X-ps-f | gggatacgccgaatcagcgagcagaaagagttttagagctagaaatagca |
| A9_pbt02X-2.1-r | agctctaaaactctttctgctcgctgattcggcgtatcccctttcagatactcgcactaa |
| E394 | cgcaggtctctgtacaggaaacagctatgac |
| E395 | gtccggtctctggccaatacgactcactataggttcc |
| ermEpbt02R-fF-f | ctcgttcgctgccacctaagaatactctacggtcacataccggaccgccgactcgggtta |
| ermEpbt02R-fF-r | gctggatcctaccaaccggcacgattgtccagcccacaacataaataatcatcccggtga |
| ermEpbt02R-fR-f | gggctggacaatcgtgccggttggtaggatccagcgggtaacaaagtgcgtaatgtccac |
| ermEpbt02R-fR-r | cgagtagttcagtagcggaaatgtcagagccagcgtcttgacggcgagcgaaccgagcgc |
| ermE-pbt02R-ps-f | aaaggggatacgctgacgcattcgatgatcgtggttttagagctagaaatagca |
| ermE-pbt02R-2.1-r | ctaaaaccacgatcatcgaatgcgtcagcgtatcccctttcagatact |
| p21-pbt02A-fF-f | ctcgttcgctgccacctaagaatactctacggtcacataccccatgtcgaggcgcacaag |
| p21_pbt02A-ps-f | tagtgcgagtatctgaaaggggatacgccgggtcctcccgccgatcccgttttagagctagaaatagca |
| p21_pbt02-2.1-r | gcgtatcccctttcagatact |
| p21_pbt02A-fR-r | cgagtagttcagtagcggaaatgtcagagccagcgtcttgccgagacgttccgccacggc |
| pbtAf3163-pRT-f | tggagttctactcccgctgaacagtcttcgcgatcccgtg |
| pbtAtr-pRT-f | ttggccttgaaatcgttagttaggctaagttgtgggctggacaatcgtgccggttggtaggatccagcgggtaacagtcttcgcgatcccgtgt |
| pbtAtr-pRT-f | acagctatgacatgattacgaattcgatctcgtggcctttctccacacg |
| pbtD-pRT3163-f | gctatgactcgttcgctgccacctaagaatatgacctggcgacgctttgacg |
| pbtD-pRT3163-r | cttagcagtatgtgaccgtagagtattaattcacatgccacctacggcgatctcgatggc |
| pbtG1-ermEp1-pSd1-f | agtgcgtggtcaccgggggctgagtatacagttgtgggctggacaatcgtgccggttggtaggatccagcgggtaccgtggcgccggcat |
| pbtG1-pSd1-r | cacaggaaacagctatgacatgattacgtcgatccccaattcacgggaacgggtgggggt |
| pbtR-ermEp1-pES120-f | ttggccttgaaatcgttagtt |
| pbtR-UNS6-SspI-r | gaccgtagagtattaatattcttaggtggcagcgaacgagtcatagcaccatcgggcgct |
| pbtR-O_ermEp1-pSd12-f | tgcgtggtcaccgggggctgagtatacagttgtgggctggacaatcgtgccggttggtaggatccagcgggtaacaaagtgcgtaatgtc |
| pbtR-O-pSd12-r | cacaggaaacagctatgacatgattacgtcgatccccaatttacctctgaatcgggattcc |
| pbtR_L92P_1.r | aaggtccggtgtaacgggcgacgtggca |
| pbtR_L92P_2.f | tgccacgtcgcccgttacaccggacctt |
| pbtR_L92P-f3 | gacccgaacgtgacccgtgccgacttcg |
| pbtR_L92P-r2 | cgaagtcggcacgggtcacgttcgggtc |
| pCRM_2-1-f | gctgggagttcgtagacggaaacaaacgcagaatccaagccatgcgctccatcaagaa |
| pCRM_1-2-r | gcttggattctgcgtttgtttccgtctacgaactcccagcgggacgtgcttggcaatca |
| pRT-overlap-r | ttagcctaactaacgatttca |
| pSETpbt1_frg1-v-f | tgccaagcttgggctgcaggtcgactctagaggttgtgggctggacaatcgtgccggttggtaggatccagcgggtaacaaagtgcgtaatgtccacctg |
| pSETpbt1_frg1-r | cccgggtcggcgagccacttctccagtt |
| pSETpbt1_frg2-f | aactggagaagtggctcgccgacccggg |
| pSETpbt1_frg2-r | aaaaccgtattctcgacttttcgctctt |
| pSETpbt1_frg3-f | aagagcgaaaagtcgagaatacggttttccgggcggcttcctcatgcttgacttgactaggataaaggggaatcagcgagcagaaagact |
| pSETpbt1_frg3-r | ttgctcatcctaccatactaggacgtgttagagcccgcacaacccgtggtcatccgccga |
| pSETpbt1_frg4-f | tgtgcgggctctaacacgtcctagtatggtaggatgagcaaacagtcttcgcgatcccgt |
| pSETpbt1_frg5-r | gctgccgatcatgaattccccgaccgcg |
| pSETpbt1_frg6-f | cgcggtcggggaattcatgatcggcagc |
| pSETpbt1_frg7-v-r | aggaaacagctatgacatgattacgtcgatccccaatattctgaactagtgtatactcagcccccggtgac |
| pSpbt(cr)-frg1-f | ggccagtgccaagcttgggctgcaggtcgactctagagcgggatggcttatgaaggattgtctcacttaggatagagcacgaggacgcttgtcggaaacg |
| pSpbt(c)n-F1-r | gaagatcctagccctcacattgatctgacagcctctataaaaaaaaggctccaaaaggagcctttaatttcacggttcccccacctcgat |
| pSpbt(c)n-F2-f | tagaggctgtcagatcaatgtgagggctaggatcttcaacactccgcctgcgagggggag |
| pSpbt(c)n-F2-r | ctggatcctaccaaccggcacgattgtccagcccacaactcagtggtcatgactgcctcc |
| pSpbt(cr)-frg3-f | ggctggacaatcgtgccggttggtaggatccagcgggtaacagtcttcgcgatcccgtgt |
| pSpbt(c)n-F3-r | ttcacacaggaaacagctatgacatgattacgaattcgattcgatccccaatattctgaactagtatcgctcgtggcctttctccacacg |

**Supplementary Table S3.** Plasmids used and generated in this study.

| Plasmid | Characteristic | Reference or source |
| --- | --- | --- |
| pbtCK02 | *pbt* cluster, Hyg^R^, EF-Tu under control of *ermE**p | Flinspach *et al.*, 2014^1^ |
| pCM4.4 | *oriT-traJ*, *ermE**p-*cas9* | Ye *et al.*, 2020^9^ |
| pES90 | *pbtR* under control of *kasO**p | This study |
| pES94 | *pbtR* under control of *kasO**p | This study |
| pES103 | *pbtA* under control of *SP44*p | This study |
| pES118 | *pbtA* under control of *actII-orf4-act1*p | This study |
| pES120 | *pbtR* under control of *ermE*p1 | This study |
| pHG5 | Contains *actII-orf4-act1*p | Gao and Smith, 2021^10^ |
| pRT801 | Integrative plasmid containing the BT1 integrase | Gregory *et al.*, 2003^11^ |
| pRTpbtA_full_ | *pbtA* under control of *ermE*p1 | This study |
| pSET152 | *oriT-traJ* | Blin *et al.*, 2016^12^ |
| pTE1707 | Insertion of *21*p upstream of *pbtA* | This study |
| pTE1708 | Insertion of *ermE*p1 upstream of *pbtR* | This study |
| pTE1709 | Insertion of *A9*p upstream of *pbtX* | This study |
| pTE1710 | *pbt* cluster lacking *pbtRG1B1O*; *ermE*p1 and *A9*p upstream of *pbtX*, *21*p upstream of *pbtA* | This study |
| pTE1711 | *pbt* cluster lacking *pbtRB1O*; *ermE*p1 and *A9*p upstream of *pbtX*, *21*p upstream of *pbtA*, *pbtG1* under control of *ermE*p1 | This study |
| pTE1712 | Expression of the *pbt* core cluster | This study |
| pTE1713 | Expression of the refactored *pbt* cluster | This study |
| pTE1718 | Expression of *3163S4* from *P. rosea* | This study |
| pTE1719 | Expression of the full-lenth *pbtA* transcript and *3163S4* from P rosea | This study |
| pTE1720 | Expression of the full-lenth *pbtA* transcript, *pbtR*, and *3163S4* from *P. rosea* | This study |
| pTE1721 | Expression of the full-lenth *pbtA* transcript, *pbtR*, *pbtD*, and *3163S4* from *P. rosea* | This study |

**Supplementary Table S4.** Inclusion list containing the precursor ions selected for fragmentation as described in the ‘LC-MS metabolomics data acquisition’ section.

| **Name** | **[M+H]^+^** | **[M+2H]^2+^** |
| --- | --- | --- |
| Peak1 | 1297.21878 | 649.113027 |
| Peak2 | 1305.24772 | 653.127498 |
| Peak3 | 1345.29046 | 673.148867 |
| Peak4 | 1327.27955 | 664.143413 |
| Peak5 | 1292.26345 | 646.635361 |
| Peak6 | 1091.15258 | 546.079929 |
| Peak7 | 1276.22131 | 638.614295 |
| Peak8 | 1108.17945 | 554.593361 |
| Peak9 | 1433.3053 | 717.156286 |
| Peak10 | 1362.26872 | 681.638 |
| Peak11 | 1178.18451 | 589.595895 |
| Peak12 | 1345.29011 | 673.148694 |
| GE2270A | 1290.26533 | 645.636305 |
| Congener D1 | 1232.22347 | 616.615373 |
| Linear Congener D2 | 1286.25516 | 643.63122 |
| Linear Congener C1 | 1290.19256 | 645.599919 |
| Congener E | 1262.23403 | 631.620655 |
| Congener C1 | 1246.23912 | 623.623198 |
| Linear Congener E/D1 | 1316.26573 | 658.636502 |
| Congener B1/C2b/D2 | 1276.24968 | 638.62848 |
| Congener B2 | 1260.25477 | 630.631023 |
| Linear CongenerB2 | 1314.28646 | 657.64687 |
| Linear Congener B1/C2b | 1330.28138 | 665.644327 |
| Congener C2a | 1306.26025 | 653.633762 |
| Linear GE2270A | 1344.29703 | 672.652152 |
| Linear Congener C2a | 1360.29194 | 680.649609 |
| Linear GE2270A 2 | 1345.30485 | 673.156065 |
| Core peptide | 1569.48433 | 785.245805 |

**AlphaPullDown analysis of PbtX co-folding with the other members of the GE2270A BGC**


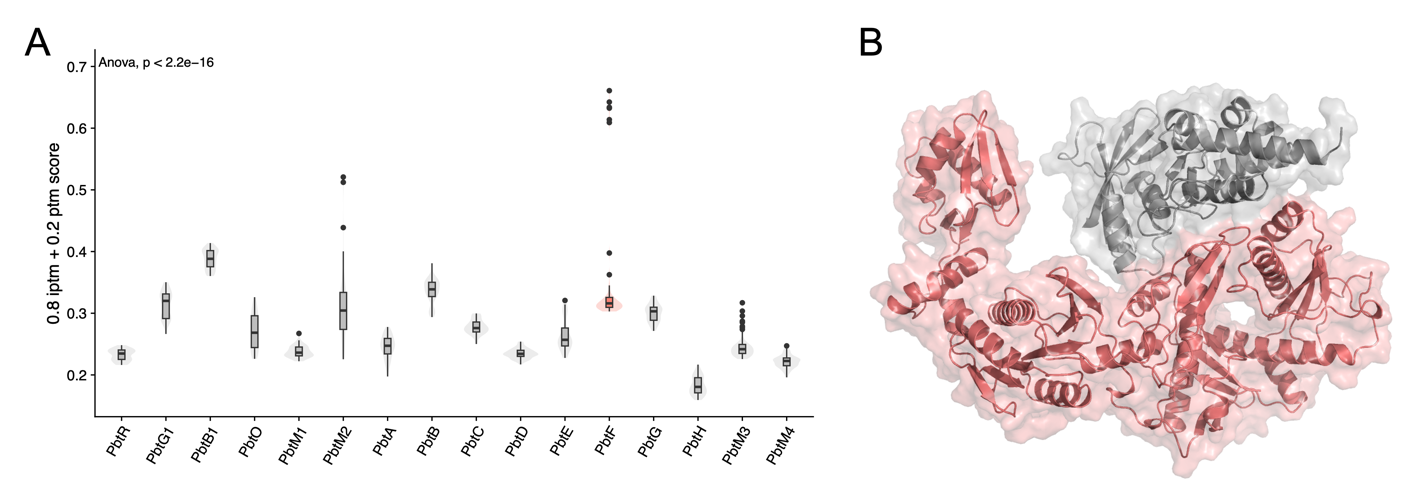


**Figure S8. AlphaPullDown analysis of PbtX co-folding with the other members of the GE2270A BGC.** A) The observed distribution of the 0.8 iPTM + 0.2 PTM scores all 50 models per each possible combination between PbtX and the other members of the GE2270A BGC. The protein that had the highest scored binding pose available (PbtF) is highlighted in red. B) The best ranked pose for PbtF (red) and PbtX (grey) which had the top 0.2 PTM + 0.8 iPTM score across all models (0.66), and the second highest PI score^13^ across all models (2.33).

**Identification of previously unreported compounds**

Among the ten features represented in the similarity network (**Fig 3C** in the paper), two previously unreported compounds are shared by Nono-Δ12, Nono-Δ12G1, and Streptomyces LW291. These compounds have molecular formulas C58H64N16O16S6 (1, calculated 1432.3010, found 1432.2994) and C55H59N15O15S6 (2, calculated 1361.2639, found 1361.2628), respectively. Both compounds appear to be linear as they share the fragment b3 (**Fig S4**) with m/z 533.0736, which corresponds to the cleavage of the amide bond between alanine 7 and thiazole D (ThzD). This fragment lacks decorations on thiazole D, thiazole E (ThzE), and asparagine (Asn), and retains the N-terminal methylketone appendage on thiazole F (ThzF), as reported by Tocchetti *et al*. (2013)^14^. The primary distinction between compounds 1 and 2 lies in the presence of an alanine residue at the C-terminal position. Specifically, compound 1 contains serine and alanine following the C-terminal proline, as seen in the parental compound GE2270A, whereas compound 2 contains only serine. This difference is supported by the presence of fragment ions y2 at m/z 274.1397 for compound 1 and m/z 203.1026 for compound 2 (**Fig S4**). Additionally, the mass spectrometry data indicate rather than the absence of decorations on the thiazoles, also the absence of the oxazoline ring and of the hydroxylation on the beta carbon of the phenylalanine residue.
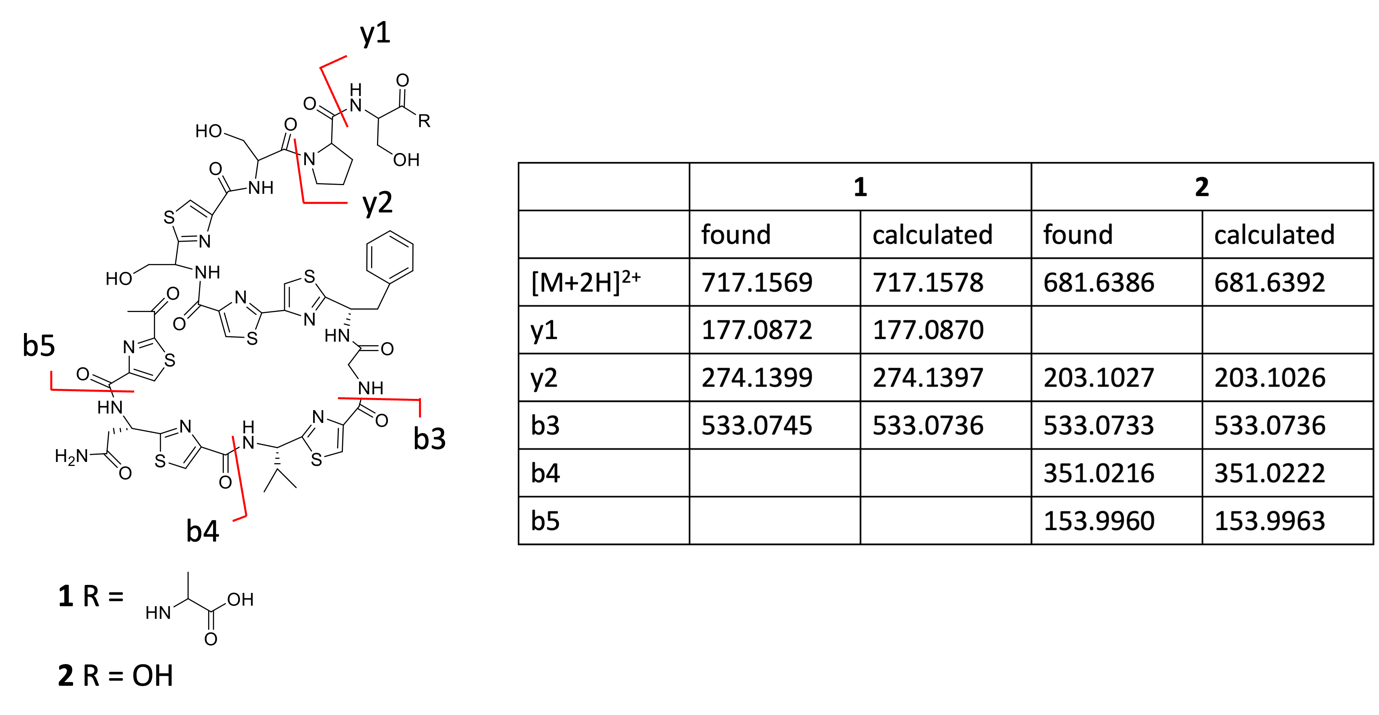


**Figure S9.** Identification of two previously unreported compounds shared by Nono-Δ12, Nono-Δ12G1, and LW291.

# Supplementary data

All supplementary data can be downloaded from here

<https://www.dropbox.com/scl/fi/re9rrke6ea1g11s94nn9u/supplementary_data.zip?rlkey=2kkrpacvy8qyusc9xmhbrvpmx&dl=0>

# Supplementary Methods

Plasmid construction

Plasmid pES90 was constructed by GenScript. The synthetised fragment with the sequence GAAGACTACTAGTGTTCACATTCGAACCGTCTCTGCTTTGACAACATGCTGTGCGGTGTTGTAAAGTCGTGGCCAGGAGAATACGACAGTCTAAGTAAGGAGTGTCCAAGTGACGAGCTCGCGCAGTAATGCCGAACGAGTGGTCCGAATCATCGACGCATCTGCGGAGCTTCTCCTCCAGAGGGGATATCGACGGGTGACCGTCGAGGAGGTCGCCAGCCATGCCGGTGTCTCCAAGAGCAGCGTCTACCTGCACTGGAACACGAAGGACGACATCTTCTACGACGCTCTCGACCGCGAGTGCGCCGCGCTCGTGTGCGAGGCCGTCGACCGCGTCAGACGCAACCCGGCCGAAATCCTGGCTCACCGGATGGCGGCCAATCTGCTCCGGATCATCCTGGACAGGCCGCTGCTGCGGGCGCTGCTGATGGGTGACCAGGCGATTCTGGGATCGCTCCGCCATGCGAAGTCGTCCGCTCTCCGATCCCGGACGGCGGCAATTGACGAGCTGATTCACCGATATCTCTCAGCGCTGCAGAAAAACCAACTCATCTGTCCCGACATCGATCTGCGCATCACCCGGAAAGCGGTGTGGGAAATGCTGCGCGGCATGATTTTCTCGGCGGGGACGGAGCCGTTGGGCAAGCCGCGCTCCGCCGAGCTGGCGCAGGTCATGACGGTTACCGTGCGGCGGGCGTTCGAGCCCAGGGGCGTACCGGGGATCGACCGGATCACCGCCGCCGCGGCCGAGGTGTTCGAGGCGTTCGACGAACTCATTCCGACCGCGGAGAACCTGGACTTCGAGCGCCCGATGGTGCTATGAACTCCATCTGGATTTGTTCAGAACGCTCGGTTGCCGCCGGGCGTTTTTTAGGATCCAGTCTTC was cloned into pUC57Kan by GenScript.

Plasmid pES94 was constructed by restriction enzyme-based cloning. The part *kasO*p*-*pbtR*-t0 was excised from pES90 using BpiI and subsequently ligated into SpeI/BamHI-digested pRT801.

Plasmid pES103 was constructed by GenScript. The synthetised fragment with the sequence ATTCCGGGGATCCGTCGACCTGTTCACATTCGAACCGTCTCTGCTTTGACAACATGCTGTGCGGTGTTGTAAAGTCTGGTGTAGGTACCAATACGACTCACTATAGGTTCCTGCTAAGGAGGCAACAAGATGAGCGAGATGGAGTTGAACCTTAACGACCTGCCCATGGACGTCTTCGAGATGGCCGACAGCGGCATGGAAGTTGAGTCACTCACCGCGGGACACGGGATGCCCGAGGTCGGTGCCTCGTGCAACTGCGTGTGCGGCTTCTGCTGTTCCTGCAGCCCGTCCGCGTGACCGTCCAGCTCGACCAGAAAAAAAAAAGCGCCGCAACTGCGGCGCTTTTTTTTTTTCGAAGCAGCTCCAGCCTACA was cloned into pUC57Kan by GenScript.

Plasmid pES118 was constructed by restriction enzyme-based cloning. The part RBS-*pbtA*-TT*_sbiB_* was amplified by PCR from pES103 using primers E395 and E394. The PCR product was digested using BsaI and ligated into NcoI/Acc65I-digested pHG5.

Plasmid pES120 was assembled by restriction enzyme-based cloning. The gBlock with the sequence GATGCAACTGGTCTCACTAGTACTGTTGTGGGCTGGACAATCGTGCCGGTTGGTAGGATCCAGCGGGTACCAATACGACTCACTATAGGTTCCTGCTAAGGAGGCAACAAAATGACGAGCTCGCGCAGTAATGCCGAACGAGTGGTCCGAATCATCGACGCATCTGCGGAGCTTCTCCTCCAGAGGGGATATCGACGGGTGACCGTCGAGGAGGTCGCCAGCCATGCCGGTGTCTCCAAGAGCAGCGTCTACCTGCACTGGAACACGAAGGACGACATCTTCTACGACGCTCTCGACCGCGAGTGCGCCGCGCTCGTGTGCGAGGCCGTCGACCGCGTCAGACGCAACCCGGCCGAAATCCTGGCTCACCGGATGGCGGCCAATCTGCTCCGGATCATCCTGGACAGGCCGCTGCTGCGGGCGCTGCTGATGGGTGACCAGGCGATTCTGGGATCGCTCCGCCATGCGAAGTCGTCCGCTCTCCGATCCCGGACGGCGGCAATTGACGAGCTGATTCACCGATATCTCTCAGCGCTGCAGAAAAACCAACTCATCTGTCCCGACATCGATCTGCGCATCACCCGGAAAGCGGTGTGGGAAATGCTGCGCGGCATGATTTTCTCGGCGGGGACGGAGCCGTTGGGCAAGCCGCGCTCCGCCGAGCTGGCGCAGGTCATGACGGTTACCGTGCGGCGGGCGTTCGAGCCCAGGGGCGTACCGGGGATCGACCGGATCACCGCCGCCGCGGCCGAGGTGTTCGAGGCGTTCGACGAACTCATTCCGACCGCGGAGAACCTGGACTTCGAGCGCCCGATGGTGCTATGAACTCCATCTGGATTTGTTCAGAACGCTCGGTTGCCGCCGGGCGTTTTTTAGGATCCGCGGCCGGAGACCATGCGTCAG, synthesised by GenScript, was digested with BsaI and combined with SpeI/NotI-digested pRT801.

Cosmid pTE1710 was constructed by HiFi DNA Assembly of five parts. The first part, comprising *pbtRB1G1O*, was amplified by PCR from pbtCK02 using primers pSETpbt1_frg1-v-f and pSETpbt1_frg2-r. The second part, comprising *pbtXM1M2*, was amplified by PCR from pbtCK02 using primers pSETpbt1_frg3-f and pSETpbt1_frg3-r. The third part, comprising *pbtABCDE*, was amplified by PCR from pbtCK02 using primers pSETpbt1_frg4-f and pSETpbt1_frg5-r. The fourth part, comprising *pbtFGHM3M4*, was amplified by PCR from pbtCK02 using primers pSETpbt1_frg6-f and pSETpbt1_frg7-v-r. The PCR products were combined with BamHI/EcoRV-digested pSET152.

Cosmid pTE1711 was constructed by HiFi DNA Assembly of two parts. The first part, comprising *pbtG1* under control of *ermE*p1, was amplified by PCR from pbtCK02 using primers pbtG1-ermEp1-pSd1-f and pbtG1-pSd1-r. The PCR product was combined with SpeI/SspI-digested pTE1710.

Cosmid pTE1712 was constructed by HiFi DNA Assembly of five parts. The first part, comprising *pbtG1B1*, was amplified by PCR from pbtCK02 using primers pSpbt(cr)-frg1-f and pSpbt(c)n-F1-r. The second part, comprising *pbtBCDE*, was amplified by PCR from pbtCK02 using primers pSpbt(c)n-F2-f and pSETpbt1_frg5-r. The third part, comprising *pbtFG* was amplified by PCR from pbtCK02 using primers pSETpbt1_frg6-f and pSpbt(c)n-F2-r. The fourth part, comprising *pbtA*, was amplified by PCR from pbtCK02 using primers pSpbt(cr)-frg3-f and pSpbt(c)n-F3-r. The PCR products were combined with BamHI/EcoRV-digested pSET152.

Cosmid pTE1713 was constructed by HiFi DNA Assembly of three parts. The first part was amplified by PCR from pbtCK02 using primers pbtR-O_ermEp1-pSd12-f and pSETpbt1_frg1-r. The second part was amplified by PCR from pbtCK02 using primers pSETpbt1_frg2-f and pbtR-O-pSd12-r. The PCR products were combined with SpeI/SspI-digested pTE1710.

Cosmid pTE1718 was constructed by HiFi DNA Assembly of two parts. The first part was amplified by PCR from *P. rosea* genomic DNA using primers 3163_S4-pRT-f and 3163_S4-pRT-r. The PCR product was combined with BamHI/EcoRV-digested pRT801.

Cosmid pTE1719 was constructed by HiFi DNA Assembly of two parts. The first part was amplified by PCR from pTE1718 using primers pbtR_L92P-f3 and 3163pbtAf-pRT-r. The second part was amplified by PCR from pRTpbtA_full_ using primers pbtR_L92P-r2 and pbtAf3163-pRT-f.

Cosmid pTE1720 was constructed by HiFi DNA Assembly of three parts. The first part was amplified by PCR from pES120 using primers pbtR-ermEp1-pES120-f and pbtR-UNS6-SspI-r. The second part was amplified by PCR from pTE1719 using primers 3163-UNS6-SspI-f and pbtR_L92P_1.r. The third part was amplified by PCR from pTE1719 using primers pRT-overlap-r/pbtR_L92P_2.f.

Cosmid pTE1721 was constructed by HiFi DNA Assembly of two parts. The first part, comprising *pbtD*, was amplified by PCR from pbtCK02 using primers pbtD-pRT3163-f and pbtD-pRT3163-r. The PCR product was combined with SspI-digested pTE1720.

Plasmid pRTpbtA_full_ was constructed by HiFi DNA Assembly of two parts. The first part was amplified by PCR from pbtCK02 using primers pbtAtr-pRT-f and pbtAtr-pRT-r. The PCR product was combined with EcoRV/SpeI-digested pRT801.

Plasmid pTE1707 was constructed by Hifi DNA assembly of five parts. The first part, comprising the upstream homology region, was amplified by PCR from pbtCK02 using primers p21-pbt02A-fF-f and pSETpbt1_frg3-r. The second part, comprising the downstream homology region, was amplified by PCR from pbtCK02 using primers p21_pbt02A-fR-r and pSETpbt1_frg4-f. The third part, comprising the protospacer, was amplified by PCR from pCM4.4 using primers p21_pbt02A-ps-f and A9_pbt02X-ps-r. The fourth part, comprising the first part of the backbone, was amplified by PCR from pCM4.4 using primers p21_pbt02-2.1-r and pCRM_2-1-f. The fifth part, comprising the second part of the backbone, was amplified by PCR from pCM4.4 using primers pbt02X-1.2-f and pCRM_1-2-r.

Plasmid pTE1708 was constructed by Hifi DNA assembly of five parts. The first part, comprising the upstream homology region, was amplified by PCR from pbtCK02 using primers ermEpbt02R-fF-f and ermEpbt02R-fF-r. The second part, comprising the downstream homology region, was amplified by PCR from pbtCK02 using primers ermEpbt02R-fR-f and ermEpbt02R-fR-r. The third part, comprising the protospacer, was amplified by PCR from pCM4.4 using primers ermE-pbt02R-ps-f and A9_pbt02X-ps-r. The fourth part, comprising the first part of the backbone, was amplified by PCR from pCM4.4 using primers ermE-pbt02R-2.1-r and pCRM_2-1-f. The fifth part, comprising the second part of the backbone, was amplified by PCR from pCM4.4 using primers pbt02X-1.2-f and pCRM_1-2-r.

Plasmid pTE1709 was constructed by Hifi DNA assembly of five parts. The first part, comprising the upstream homology region, was amplified by PCR from pbtCK02 using primers A9-pbt02X-fF-f and A9_pbt02X-fF-r. The second part, comprising the downstream homology region, was amplified by PCR from pbtCK02 using primers A9_pbt02X-fR-f and A9_pbt02X-fR-r. The third part, comprising the protospacer, was amplified by PCR from pCM4.4 using primers A9_pbt02X-ps-f and A9_pbt02X-ps-r. The fourth part, comprising the first part of the backbone, was amplified by PCR from pCM4.4 using primers A9_pbt02X-2.1-r and pCRM_2-1-f. The fifth part, comprising the second part of the backbone, was amplified by PCR from pCM4.4 using primers A9_pbt02X-1.2-f and pCRM_1-2-r.

# References

1. Flinspach, K., Kapitzke, C., Tocchetti, A., Sosio, M. & Apel, A. K. Heterologous Expression of the Thiopeptide Antibiotic GE2270 from Planobispora rosea ATCC 53733 in Streptomyces coelicolor Requires Deletion of Ribosomal Genes from the Expression Construct. *PLoS One* **9**, e90499 (2014).

2. Cobb, R. E., Wang, Y. & Zhao, H. High-Efficiency Multiplex Genome Editing of *Streptomyces* Species Using an Engineered CRISPR/Cas System. *ACS Synth Biol* **4**, 723–728 (2015).

3. van Wezel, G. P., Takano, E., Vijgenboom, E., Bosch, L. & Bibb, M. J. The tuf3 gene of Streptomyces coelicolor A3(2) encodes an inessential elongation factor Tu that is apparently subject to positive stringent control. *Microbiology (N Y)* **141**, 2519–2528 (1995).

4. Olsthoorn-Tieleman, L. N., Palstra, R.-J. T. S., van Wezel, G. P., Bibb, M. J. & Pleij, C. W. A. Elongation Factor Tu3 (EF-Tu3) from the Kirromycin Producer *Streptomyces ramocissimus* Is Resistant to Three Classes of EF-Tu-Specific Inhibitors. *J Bacteriol* **189**, 3581–3590 (2007).

5. Del Carratore, F. *et al.* Multi-omics Study of Planobispora rosea, Producer of the Thiopeptide Antibiotic GE2270A. *mSystems* **6**, (2021).

6. MacNeil, D. J. *et al.* Analysis of Streptomyces avermitilis genes required for avermectin biosynthesis utilizing a novel integration vector. *Gene* **111**, 61–68 (1992).

7. Bennett, P. M., Grinsted, J. & Richmond, M. H. Transposition of TnA does not generate deletions. *Mol Gen Genet* **154**, 205–211 (1977).

8. Gomez‐Escribano, J. P. & Bibb, M. J. Engineering *Streptomyces coelicolor* for heterologous expression of secondary metabolite gene clusters. *Microb Biotechnol* **4**, 207–215 (2011).

9. Ye, S., Enghiad, B., Zhao, H. & Takano, E. Fine-tuning the regulation of Cas9 expression levels for efficient CRISPR-Cas9 mediated recombination in *Streptomyces*. *J Ind Microbiol Biotechnol* **47**, 413–423 (2020).

10. Gao, H. & Smith, M. C. M. Use of orthogonal serine integrases to multiplex plasmid conjugation and integration from E. coli into Streptomyces. *Access Microbiol* **3**, (2021).

11. Gregory, M. A., Till, R. & Smith, M. C. M. Integration Site for *Streptomyces* Phage φBT1 and Development of Site-Specific Integrating Vectors. *J Bacteriol* **185**, 5320–5323 (2003).

12. Blin, K., Pedersen, L. E., Weber, T. & Lee, S. Y. CRISPy-web: An online resource to design sgRNAs for CRISPR applications. *Synth Syst Biotechnol* **1**, 118–121 (2016).

13. Malhotra, S., Joseph, A. P., Thiyagalingam, J. & Topf, M. Assessment of protein–protein interfaces in cryo-EM derived assemblies. *Nat Commun* **12**, 3399 (2021).

14. Tocchetti, A. *et al.* Capturing Linear Intermediates and C-Terminal Variants during Maturation of the Thiopeptide GE2270. *Chem Biol* **20**, 1067–1077 (2013).
